# Amphibian cellular immune response to chytridiomycosis at metamorphic climax

**DOI:** 10.1007/s12026-025-09599-5

**Published:** 2025-01-30

**Authors:** Josephine E. Humphries, Allan Hicks, Chantal Lanctôt, Hamish McCallum, David Newell, Laura F. Grogan

**Affiliations:** 1 School of Environment and Science, Griffith University, Southport, QLD 4222 Australia; 2 Centre for Planetary Health and Food Security, Griffith University, Southport, QLD 4222 Australia; 3 School of Pharmacy and Medical Sciences, Griffith University, Southport, QLD 4222 Australia; 4 Australian Rivers Institute, Griffith University, Southport, QLD 4222 Australia; 5 Faculty of Science and Engineering, Southern Cross University, Lismore, NSW 2480 Australia; 6 School of the Environment, University of Queensland, St Lucia, QLD 4067 Australia

**Keywords:** Amphibian, Metamorphosis, *Batrachochytrium dendrobatidis*, Disease, Histology, Immune cells

## Abstract

The fungal disease chytridiomycosis (caused by *Batrachochytrium dendrobatidis* [Bd]) is a primary contributor to amphibian declines. The frog metamorphic stages, characterised by extensive physiological reorganisation and energy expenditure, have heightened susceptibility to Bd. However, little is known about how these metamorphic stages respond immunologically to Bd infection. In this study, we examined Bd infection and the cellular immune response of *Mixophyes fleayi* at Gosner stages 40, 42 and 45, using blood smears and skin and liver histology. Although proportional differences were observed, the impact of Bd exposure appeared negligible prior to Gosner stage 45 (onset of morbidity), with no significant differences observed in absolute leukocyte counts for blood or liver samples between control and Bd-exposed groups at Gosner stages 40 and 42. Animals exhibiting clinical signs at Gosner stage 45 demonstrated significant elevation in liver leukocyte counts, blood neutrophil and monocyte counts and neutrophil-to-lymphocyte ratios. These findings are reminiscent of the amplified inflammatory response characteristic of immunopathology in clinically infected amphibians. Interestingly, a subset of exposed animals that had apparently cleared infections at Gosner stage 45 had similar blood leukocyte counts but reduced liver leukocyte counts compared to naïve controls. This could be a consequence of prior cellular consumption during pathogen removal or effective immune regulation via anti-inflammatory protective feedback mechanisms. We recommend targeted gene expression analyses (e.g. immunomodulatory cytokines) to establish the mechanisms responsible for the varied immune expression and infection outcomes across metamorphosis.

**Supplementary Information:**

The online version contains supplementary material available at 10.1007/s12026-025-09599-5.

## Introduction

The fungal disease chytridiomycosis (caused by the pathogenic fungus *Batrachochytrium dendrobatidis*, hereafter Bd [1]) is a primary contributor to the declines observed in amphibian species globally [2]. Chytridiomycosis is the greatest infectious disease threat to vertebrate biodiversity, with at least 90 amphibian extinctions putatively attributed to Bd infection [3]. Bd infects amphibian skin and produces clinical pathology such as loose adherence of stratum corneum [4], hyperkeratosis (thickened outer layer) and hyperplasia (increased cell production) [5]. Animals with clinical Bd infection have impaired electrolyte transport and osmoregulatory function, culminating in cardiac arrest and mortality [6]. The mechanisms by which amphibian hosts can defend themselves against the fungus have been the focus of a great number of studies to date (reviewed by [7, 8]). However, most studies have examined the post-metamorphic stages of frog development, in part due to the heightened disease-associated mortality of post-metamorphic animals compared to larval Amphibia [9, 10]. This has resulted in a gap in our understanding of how the pro-metamorphic (Gosner stages 36–41) and metamorphic climax (Gosner stages 42–46) stages respond to Bd [11, 12], limiting our understanding of infection dynamics at a population scale and hence reducing our capacity to protect populations from decline.

The extensive reorganisation that characterises the metamorphic transition likely influences the susceptibility of hosts to various stressors [11]. Metamorphosis involves the rearrangement of larval-specific structures into adult cells and tissues [13–15], including those associated with the immune system [16, 17]. Metamorphic reorganisation is primarily controlled by thyroidal hormonal expression, with the peak of these hormones recorded at metamorphic climax [18]. This restructuring, combined with the extensive energy requirements of this transformation [19], likely impacts the capacity of metamorphic animals to mount a successful immune response. We previously demonstrated the significance of the metamorphic transition for Bd-associated susceptibility in amphibians [12], and suggested that the metabolic dysregulation we observed in clinically infected animals may be exacerbated by immune system dysfunction at metamorphic climax [20]. Aspects of the immune system may thus be impaired at the peak of metamorphic reorganisation (climax), and this may, in turn, reduce the capacity of animals to avoid Bd-induced pathology.

While the skin is the primary site of Bd infection in Amphibia, other organs and systems such as the liver, spleen and blood play important roles in determining the outcome of infection. For example, the liver is a site of amphibian haematopoiesis and a major immunological organ, contributing to the early stages of the inflammatory response [21, 22] and the differentiation of leukocytes [23]. Exposure to physiological stressors (e.g. pollutants) has been demonstrated to stimulate the infiltration of inflammatory cells into the amphibian liver [24, 25]. Similarly, liver melanomacrophage aggregation has been positively associated with exposure to stressors [26, 27], with animals exposed to environmental pollutants displaying a greater abundance of melanomacrophages than controls [26, 28–30]. Hepatic macrophages have phagocytic properties and have been shown to contribute to cell clearance [31], liver detoxification and immune defence in amphibians [27, 32, 33], with important roles in immune system regulation via the production of inflammatory mediators. Indeed, melanomacrophage aggregate abundance is often used as a biomarker for detecting liver damage [34] and toxicity [27, 28, 35]. The presence of vacuoles (small circular structures void of staining) in amphibian livers represents hepatocyte degeneration and fluid accumulation [36], with liver vacuolisation identified in Bd-infected [37] and carbonyl-treated [28] animals. Consistent with these reported responses to physiological stressors, both leukocyte infiltration and melanomacrophage cell area in the liver were found to be positively correlated with the development of clinical signs of chytridiomycosis, indicating an active (if ineffective) inflammatory process [37]. Other studies have shown that Bd infections reduced splenic lymphocyte responses and decreased white blood cell concentrations in common green (*Litoria caerulea*) and white-lipped (*Litoria infrafrenata*) tree frogs [38]. Inhibition of the lymphocyte proliferation response has been linked with Bd inhibitory factors [39], and such suppression of the adaptive immune response to Bd likely contributes to the development of immunopathology [7].

Circulating blood leucocytes are also an important component of the host immune defence against pathogenic invasion, and the relative frequencies of leukocytes can be used to classify the host response. For example, the innate immune response is typically characterised by elevated neutrophils, while elevated lymphocytes are more characteristic of the adaptive immune response [40]. The varied proportions of infiltrating cells can also denote immune functionality. For example, the relationship between the number of neutrophils and lymphocytes (N/L ratio) is a known disease biomarker that communicates information about the innate and adaptive components of the immune response [41]. Elevated N/L ratios are indicative of the pro-inflammatory state [41]. Previous studies have separately established the impact of Bd infection on circulating leukocytes [42] and throughout metamorphic development [43]. However, the impacts of Bd exposure on immune cell composition in immunoreactive tissues and circulating blood leukocytes have not been examined throughout metamorphosis. We, therefore, evaluated the interactive effects of metamorphic immune reorganisation and the Bd-induced immune response by combining circulating blood leukocyte counts and histological visualisation of skin and liver tissues at several time points throughout amphibian metamorphosis.

## Methodology

All methods were conducted according to approved guidelines with ethical approval and permits for the use of animals (Griffith University Animal Ethics Committee permit [ENV/03/20/AEC] and Queensland scientific permit [P-PTUKI-100045853]) and *Batrachochytrium dendrobatidis* (Restricted matter permit PRID000746). A more detailed explanation of the experimental methodology can be found in [12].

### Animal husbandry and experimental exposures.

We collected a partial egg clutch of Fleay’s barred frog (*Mixophyes fleayi*) from Dalrymple Creek Goomburra Main Range National Park S 27° 58.878′ E 152° 21.101′ (− 27.981300, 152.351683) and transferred them to a lab at Griffith University. We reared the hatched *M. fleayi* naïve to Bd for ~ 340 days until a subgroup (*n* = 78) reached the pre-metamorphic stage (Gosner stages 31–35). Husbandry conditions were maintained to approximate *M. fleayi’s* natural environment (23–25 °C and 12:12-h light/dark cycle). We acclimated a subgroup of animals (78 animals at Gosner stage 35) to individual enclosures for 1 week prior to Bd exposures. Experimental tadpoles were housed in aquaria (2.5 L) containing 2-L artificial soft water [44] aerated with air stone bubblers, and they were fed Sera Micron Nature Fry Food in proportion to their body weight (2% weight every 2 days). We did not feed animals shortly before or during experimental exposures. As animals progressed to Gosner stage 42 (forelimbs emerging), they were moved to semi-terrestrial metamorph enclosures with PVC shelters and areas of dry gravel substrate for burrowing. We produced a Bd inoculant using the isolate DalrympleCreek-Fleayi-21-JH-1-p3 [12]. We exposed 39 tadpoles to the Bd isolate (3-mL inoculant [10^6^ zoospores/mL] and 30-mL soft water) and 39 to 33-mL soft water only (sham-exposed controls) for 8 h. We standardised husbandry between individuals and used strict hygiene procedures throughout the experiment.

Following exposures, animals were monitored daily, and physiological measurements (including skin swab sampling for quantification of Bd loads) were performed weekly [12]. We measured Bd loads via quantitative polymerase chain reaction (qPCR) using previously described methods [45, 46]. Detailed methodology is provided in [12]. Any animals displaying clinical signs of chytridiomycosis were euthanised immediately via double pithing and decapitation in accordance with ethical regulations. Subgroups of control and exposed animals were selected using a randomised block design at Gosner stages 40 (6 control, 5 exposed), Gosner stage 42 (6 control, 5 exposed) and Gosner stage 45 (13 control, 29 exposed [21 opportunistically sampled when they developed overt clinical signs of disease, and 8 animals sampled without clinical signs at experiment termination]). All animals were skin swabbed for Bd, and selected animals were humanely euthanised, after which blood was extracted immediately via cardiocentesis with heparinised microhematocrit tubes to prevent clotting. Sections of liver and skin tissues were then sampled post-mortem.

Up to five blood smears were generated for each animal and allowed to air dry before fixation in 100% methanol. We stained blood smears with Wright’s stain to enable the differentiation of blood cells using eosin and methylene blue dyes. Sections of sampled skin and liver tissues were fixed in 10% neutral buffered formalin for 2 h before being transferred for storage in 70% ethanol [47]. The skin sections for animals at Gosner stage 40 were tadpole mouthparts (where Bd infection occurs), whereas, for stages 42 and 45, we collected ventral abdominal skin. We dehydrated tissue samples using a graded ethanol series before immersion in xylene. We then embedded samples in paraffin wax (orienting tissues to enable transverse sectioning) before transfer to embedding moulds. The embedded samples were cut into 5-µm-thick tissue sections using a rotary microtome before mounting them onto microscope slides. Multiple sections were produced for each animal to enable varied staining procedures and subsequent analyses. Mounted slides were deparaffinised and rehydrated before staining with either haematoxylin and eosin (H&E) or toluidine blue.

### Cell counts in QuPath

We scanned entire stained microscopy slides (40 ×) using an Olympus SLIDEVIEW VS200 high-throughput bright-field slide scanner. Photomicrographs were taken for each specimen to enable visualisation and digital analyses of the scanned slides using QuPath software (v0.4.3). QuPath (abbreviation of Quantitative Pathology) is an open-source bioimage analysis software for digital pathology and whole-slide image analysis [48]. QuPath is one of the most widely used bioimage analysis software packages available globally [49] and is often used for quantitative analyses of tissue pathology [34, 50–52]. A detailed description of the QuPath workflow and cell counts used in the present study is provided in supplementary materials (4.2 and 4.3, respectively). Only successfully processed specimens were analysed, and only Fields of View (FOV; 250 × 250 µm) containing cells and tissue structure without obvious damage or impurity were included in the analyses. The number of specimen samples included in the statistical analyses of H&E and toluidine blue-stained histology samples and Wright’s-stained blood smears are shown in supplementary materials (Supp. Table S1).

Cell counts involved the manual and semi-automated detection of immune cells using whole-slide imaging (Supp. Fig. S3-6). We used automated cell detection to identify cells using nuclei optical density with specified baseline features (cell radius, background intensity, etc.; Supplementary materials S3). We checked the validity of initial cell annotations and adjusted annotation criteria accordingly. We then manually classified subgroups of individual cell types (e.g. white blood cells) and used these to train object classifiers and expand classifications across selected areas. This process was repeated until all cells were reliably classified across 20 randomly selected FOVs. Liver cell counts were performed on all 20 FOVs statistically randomised within a grid instead of one larger section to ensure representation of the entire tissue section. This allowed us to measure immune cell counts as a sum of a specified area (5 mm^2^) for all specimens.

We measured immune cell abundance in toluidine blue-stained liver histological samples by performing white (WBC) and red (RBC) blood cell counts for 20 FOVs statistically randomised within a grid (250 × 250 µm) per specimen (5 mm^2^) (Supp. Fig. S3). We used semi-automated cell detection described above to identify cells using nuclei optical density. We classified cell types (hepatocytes, RBC, WBC and melanomacrophage aggregations) and used these to train object classifiers and expand classifications across all 20 FOVs. There were some caveats to the chosen methodology; for instance, it should be noted that hepatocyte nuclei counts do not represent individual cells as hepatocytes can have multiple nuclei. Additionally, the varied size of melanomacrophage aggregates necessitated the additional measurement of melanomacrophage density by measuring the pigmented cell areas and calculating the sum area per 5 mm^2^ measured (Supp. Fig. S6), using similar methodologies to those outlined by Awaad et al. [53, 54].

For histological skin analyses, we could not reliably identify immune cells within the skin tissues sampled (Fig. 7, Supp. Fig. S7). As immune cell recruitment to the skin of Bd-infected animals is often inconsistent within and between species [7], skin samples were manually assessed for Bd-induced pathology [55]. Classifications were made based on the abundance of Bd zoosporangia, the extent of hyperkeratosis and skin sloughing and overall damage to epidermal and dermal layers. The severity of Bd infection was manually rated on a scale of 0–5, and scores were compared between exposure groups across development. For Wright’s-stained blood smears, we counted blood cells from 20 FOVs (5 mm^2^) statistically randomised within a grid (250 × 250 µm). We manually classified and counted each blood leukocyte type (monocytes, lymphocytes, neutrophils, basophils, eosinophils and thrombocytes; Supp. Fig. S8-9) for each selected FOV following [56]. This enabled the calculation of differential blood leukocyte counts, total cell counts and neutrophil-to-lymphocyte (N/L) ratios.

### Data analyses

All statistical analyses were performed in R version 4.3.1 [57]. For the Wright’s-stained blood smears, relative counts of each blood leukocyte type were calculated per 3000 RBCs (calculated based on average RBC counts per sample to account for differences in smear and blood volume characteristics, Supp. Table S2), and counts were log-transformed (log_10_[count + 1]) to approximate normal distributions. We performed one-way multivariate analyses of variance (MANOVA) for Wright’s-stained blood smears, with univariate post hoc analyses for each cell type (Supp. Table S3-7). The log-transformed relative counts of each leukocyte (monocyte, lymphocyte, neutrophil, basophil, eosinophil and thrombocyte) were the (six) response variables, and Gosner stage (40, 42 and 45) and Bd infection (control, exposed-cleared and exposed-moribund) were the predictor variables. We performed one-way MANOVAs to compare the log-transformed leukocyte counts of control animals throughout development (Gosner stages 40, 42 and 45, Supp. Table S3) and between exposure groups (control, exposed-cleared and exposed-moribund) at each Gosner stage (Supp. Table S4-7).

For toluidine blue-stained liver histological samples, we performed one-way analyses of variance (ANOVA) to compare the counts of each cell type (WBC, RBC and melanomacrophage) between the Gosner stages of control animals (Supp. Table S9-11). We also performed individual *t*-tests to compare the cell counts (WBC, RBC and melanomacrophage) of control and exposed livers at Gosner stages 40 and 42 (Supp. Table S12-13) and one-way ANOVAs to compare infection outcomes (control, exposed-cleared and exposed-moribund, Supp. Table S14-16) at Gosner stage 45. We statistically compared melanomacrophage aggregate density (the area occupied by melanomacrophage aggregations) of control animals throughout development (one-way ANOVA, Supp. Table S17) and infection outcome groups at Gosner stage 45 (one-way ANOVA, Supp. Table S18). Following Shapiro–Wilk normality tests, we transformed cell counts (log_10_ or square root) to meet the assumption of normality for ANOVA analyses and conducted Tukey HSD post hoc tests for all significant ANOVA outcomes.

## Results

In the same study animals, we have previously demonstrated the dynamics of Bd infection throughout *Mixophyes fleayi* metamorphic development, highlighting a plateau in infection loads at Gosner stage 40 followed by temporary infection clearance at Gosner stage 42 [12]. At Gosner stage 45, a subgroup of exposed animals was opportunistically sampled at the onset of morbidity (*n* = 21/29). Of the exposed animals that survived until the termination of the experiment (*n* = 8), the majority had apparently cleared Bd infections (*n* = 7/8) despite having high infection burdens in the preceding weeks [12]. We thus included ‘infection outcome’ (negative control, exposed-cleared and exposed-moribund animals) as a variable in ad hoc analyses for Gosner stage 45 samples. The only exposed animal that had detectable Bd loads at Gosner stage 45 but was not displaying clinical signs (B25; Supp. Fig. S1−2) was included in the cleared subgroup.

**Fig. 1:**
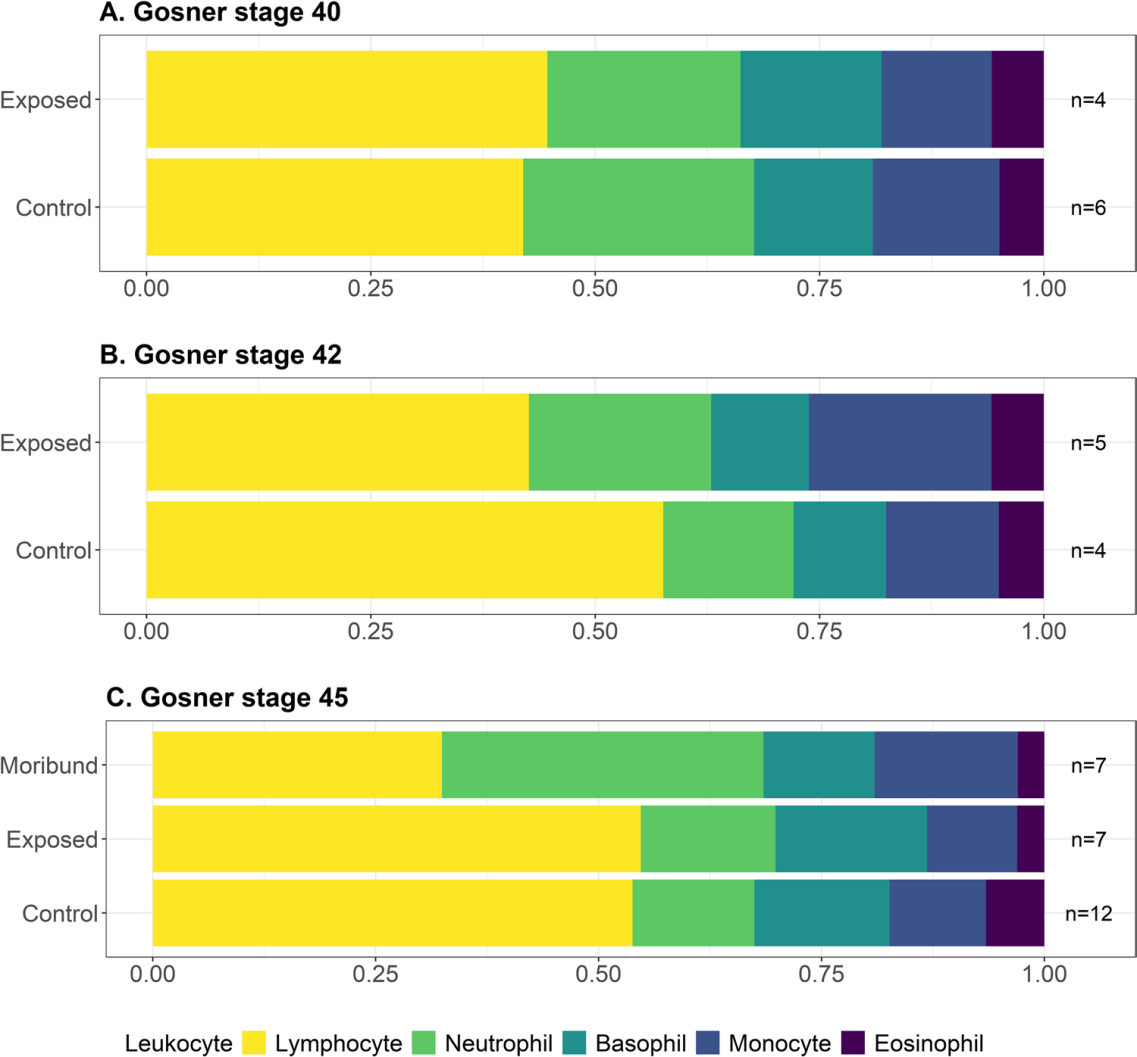
Proportional blood leukocyte counts for Gosner stages 40 (**A**, pre-metamorphosis), 42 (**B**, the onset of metamorphic climax) and 45 (**C**, the final stage of metamorphic climax) and sample group (control, exposed-cleared and exposed-moribund). Sample sizes for each sample group are indicated as *n* values. Leukocyte counts were performed using blood smears stained with Wright’s stain, and values were calculated as a proportion of the total leukocyte counts

The proportional blood cell counts of control and exposed groups were consistent at Gosner stage 40. In contrast, at Gosner stage 42, the proportion of neutrophils was elevated. Lymphocytes were reduced in exposed compared to control animals (Fig. 1). At Gosner stage 45, leukocyte proportions were similar between control and exposed-cleared groups, but neutrophils and monocytes were elevated in moribund animals (Fig. 1). Likewise, the neutrophil-to-lymphocyte (N/L) ratios of control and exposed groups were comparable at Gosner stage 40, elevated for exposed animals at Gosner stage 42 and substantially higher in Gosner stage 45 moribund animals compared to control or exposed-cleared groups (Supp. Fig. S11).

**Fig. 2:**
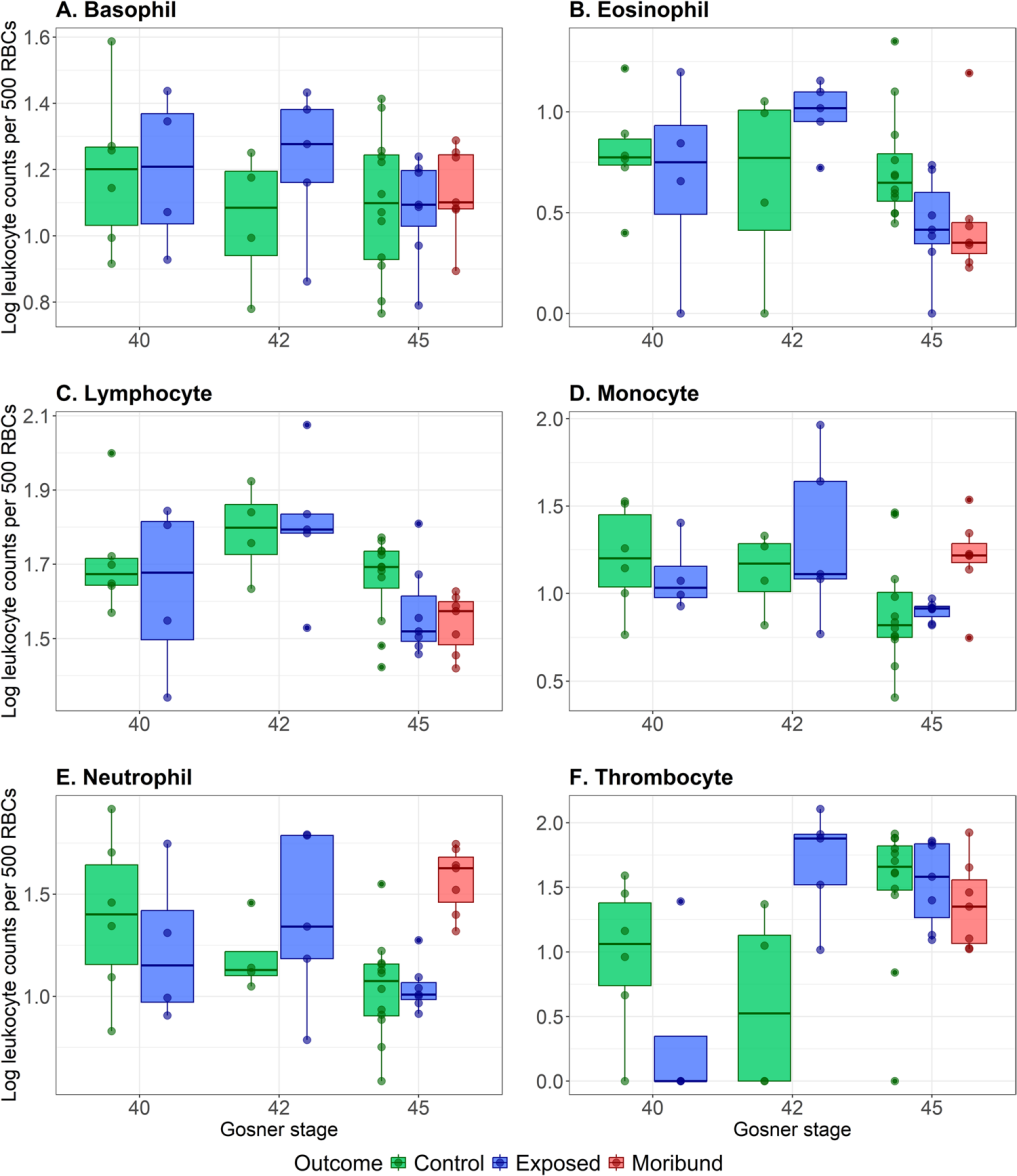
Log-transformed blood cell counts for basophils (**A**), eosinophils (**B**), lymphocytes (**C**), monocytes (**D**), neutrophils (**E**) and thrombocytes (**F**), from 20 randomly selected FOVs (250 × 250 µm) per specimen (5 mm.^2^). Counts are coloured by sample group (control, exposed-cleared and exposed-moribund) and development stage; Gosner stages 40 (pre-metamorphosis, *n* = 11), 42 (the onset of metamorphic climax, *n* = 11) and 45 (the final stage of metamorphic climax, *n* = 42). Cell counts were performed using blood smears stained with Wright’s stain. Boxplots show the interquartile range (column), median (horizontal line), minimum and maximum values excluding outliers (whiskers)

The blood leukocyte counts of control animals did not significantly differ between Gosner stages (Pillai’s Trace = 0.655, *p*-value = 0.316, one-way MANOVA, Supp. Table S3), as demonstrated in Fig. 2 and Supplementary Fig. S12. We did not detect a significant impact of Bd exposure on the blood leukocyte counts of animals sampled at Gosner stages 40 or 42 (one-way MANOVAs for individual Gosner stages, Supp. Table S4-5). However, blood leukocyte counts differed significantly between infection outcome groups (control, exposed-cleared and exposed-moribund) at Gosner stage 45 (Pillai’s Trace = 0.902, *p*-value = 0.0125, Supp. Table S6). The differences in infection outcome cell counts were predominantly driven by monocytes (*F*_2,23_ = 3.85, *p*-value = 0.0362) and neutrophils (*F*_2,23_ = 18.0, *p*-value < 0.0001), although lymphocytes and eosinophils were also near significant (Supp. Table S7). Both monocytes and neutrophils were elevated in animals sampled at the point of morbidity in comparison to control and cleared groups (Fig. 2). In contrast, counts of lymphocytes and eosinophils were reduced in exposed-cleared and -moribund animals in comparison to controls (Fig. 2).

**Fig. 3:**
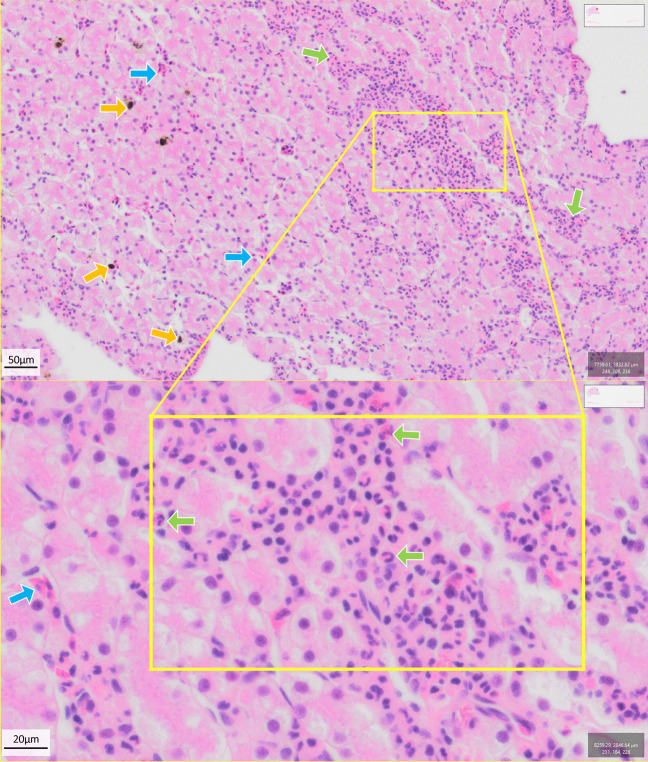
Example of a typical liver histological sample from a negative control animal (*Mixophyes fleayi*) at Gosner stage 40 (H&E stain) showing immune cells (green arrows), erythrocytes (blue arrows) and melanomacrophage assemblages (yellow arrows). Images were taken from QuPath digital pathology and image analysis software [48]. Scale bar = 50 µm (top) and 20 µm (bottom)

**Fig. 4:**
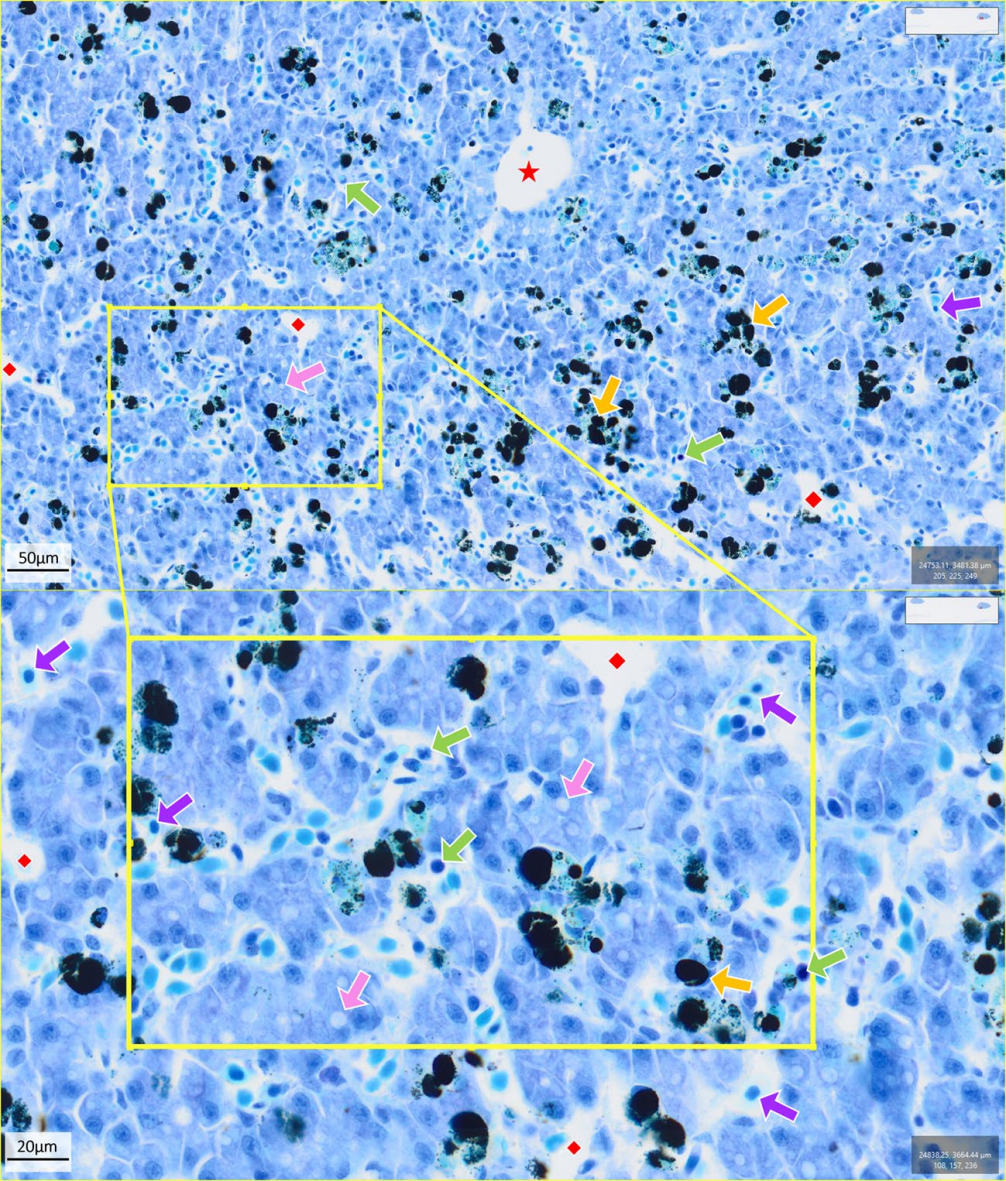
Example of a typical liver histological sample from a moribund animal (*Mixophyes fleayi*) at Gosner stage 45 (toluidine blue stain) showing immune cells (green arrows), erythrocytes (purple arrows), melanomacrophage assemblages (yellow arrows), vacuolated cells (pink arrows), sinusoids (red diamond) and blood vessels (red star). Images were taken from QuPath digital pathology and image analysis software [48]. Scale bar = 50 µm (top) and 20 µm (bottom)

We examined the effect of Bd infection on amphibian livers using histological samples stained with H&E and toluidine blue (see example slides in Figs. 3 and 4, respectively), quantifying the abundance (cell counts per 5 mm^2^) of hepatocytes, erythrocytes, melanomacrophages and leukocytes within liver samples (Supp. Fig. S3-6).

**Fig. 5:**
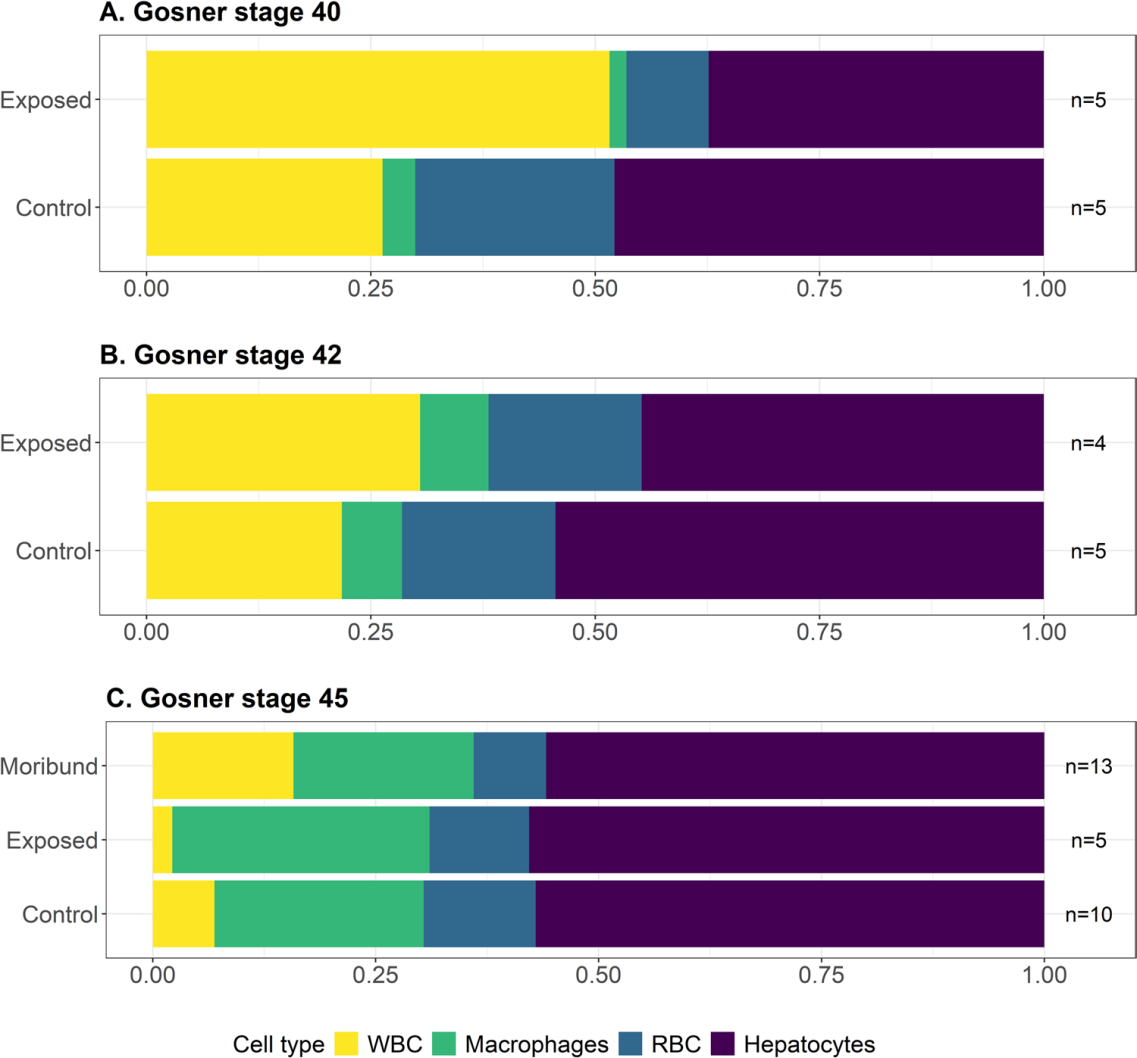
Proportional blood cell counts of white blood cells (WBC), hepatocytes and melanomacrophages between Gosner stages 40 (**A**, pre-metamorphosis), 42 (**B**, the onset of metamorphic climax) and 45 (**C**, the final stage of metamorphic climax) and sample group (control, exposed-cleared or exposed-moribund animals). Sample sizes for each sample group are indicated as *n* values. Cell counts were performed using histological liver samples stained with toluidine blue and values were calculated as a proportion of the total cell counts

Proportional leukocyte counts (WBC) were elevated in the livers of Bd-exposed animals at Gosner stages 40 and 42, and in moribund animals at Gosner stage 45, compared to both control and exposed-cleared groups (Fig. 5). The cleared subgroup, at Gosner stage 45, had the lowest proportion of leukocytes of any sample group, including controls.

**Fig. 6:**
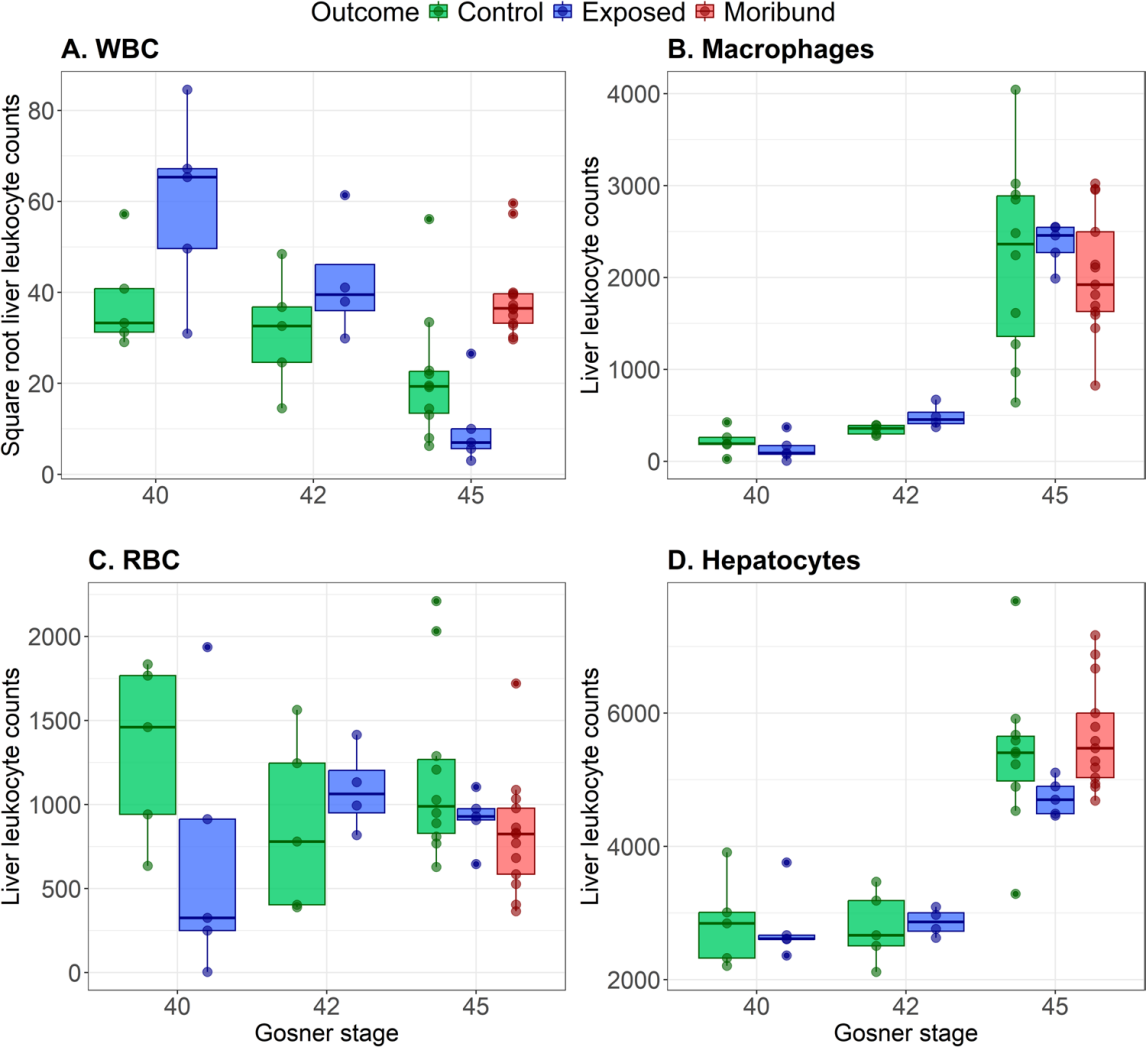
Blood cell counts of white blood cells (WBC; **A**), melanomacrophages (**B**), red blood cells (RBC; **C**) and hepatocytes (**D**) from 20 randomly selected FOVs (250 × 250 µm) per specimen (5 mm.^2^). Counts are coloured by sample group (control, exposed-cleared and exposed-moribund) and development stage; Gosner stages 40 (pre-metamorphosis, *n* = 11), 42 (the onset of metamorphic climax, *n* = 11) and 45 (the final stage of metamorphic climax, *n* = 42). Cell counts were performed using histological liver samples stained with toluidine blue. Boxplots show the interquartile range (column), median (horizontal line), minimum and maximum values excluding outliers (whiskers)

The white blood cell (WBC) counts of control animal livers did not significantly differ between Gosner stages 40, 42 and 45 (one-way ANOVA, Supp. Table S9, Supp. Fig. S16), as demonstrated in Fig. 6. Melanomacrophage abundance increased significantly throughout development (*F*_2,17_ = 15.28, *p*-value = 0.000159, Supp. Table S11) to peak at Gosner stage 45 (Fig. 6). To account for the varied area occupied by melanomacrophage aggregations, we also compared melanomacrophage area (density; Supp. Table S17-18). We found significant differences in macrophage density between Gosner stages (*F*_2,17_ = 28.44, *p*-value < 0.0001) with a peak at Gosner stage 45 (Supp. Fig. S18).

Comparisons of liver cell abundance (WBC, RBC and macrophages) between control and exposed groups at each Gosner stage (*t*-tests for exposure groups, Supp. Table S12-13) highlighted the limited impact of Bd exposure for any cell type at Gosner stages 40 and 42 (Fig. 6). When comparing cell counts between infection outcome groups at Gosner stage 45 (one-way ANOVA for infection outcome), we did not detect significant differences for RBCs or melanomacrophages (Supp. Table S15-16, S18). White blood cell abundance varied at Gosner stage 45 (*F*_2,25_ = 17.4, *p*-value < 0.0001), with significant differences demonstrated between all infection outcome groups (Tukey HSD, Supp. Table S14). White blood cell counts were elevated in exposed-moribund animals and decreased in exposed-cleared animals to levels lower than same-stage controls (Fig. 6).

**Fig. 7:**
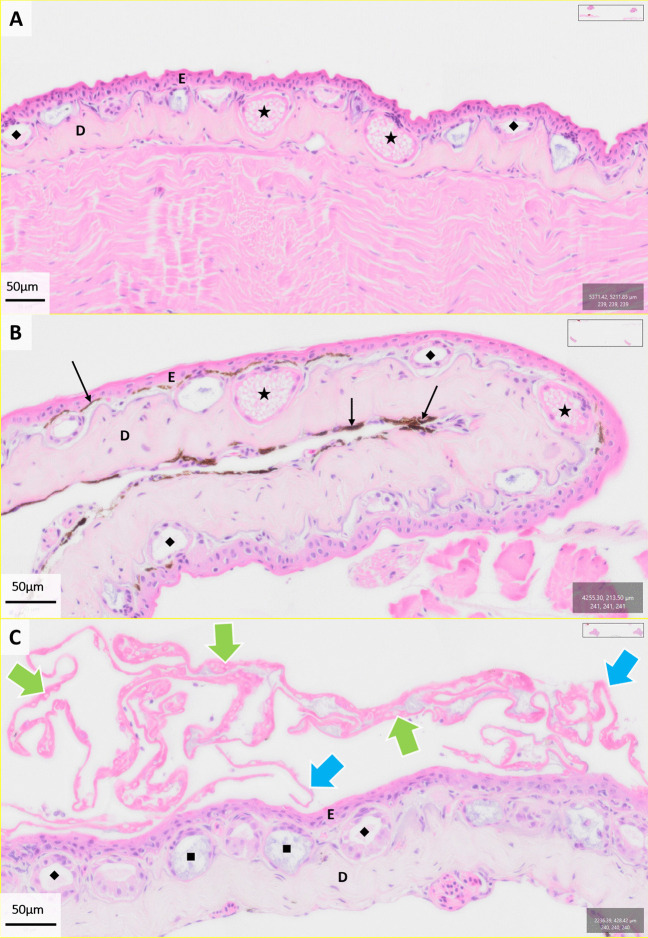
Examples of Bd-associated pathology of H&E-stained skin histological samples from control (**A**), exposed-cleared (**B**) and exposed-moribund (**C**) *Mixophyes fleayi* sampled at Gosner stage 45. Structural features are shown with epidermal (**E**) and dermal (**D**) layers, melanin pigmentation (black arrows), serous/granular (star) and mucosal (diamond) glands. The heavy infection burden (Bd zoosporangia, green arrows) and extensive skin shedding (hyperkeratosis, blue arrow) are identified in samples from moribund animals (C). Images were taken from QuPath digital pathology and image analysis software [48]. Scale bar = 50 µm (A, B and C)

We compared skin samples histologically across infection groups (control, exposed-cleared and exposed-moribund) for evidence of Bd-associated pathology (e.g. structural differences, presence of zoosporangia) at Gosner stages 40, 42 and 45 (Fig. 7). We only identified histological evidence of Bd and associated pathology (Bd zoosporangia, hyperkeratosis and skin sloughing and overall damage to epidermal and dermal layers) in exposed animals demonstrating clinical signs (exposed-moribund group) at Gosner stage 45 (Fig. 6). Despite detectable Bd loads via qPCR (mean and SD = 5.41 ± 2.70 log_10_[ITS copies per swab + 1]) [12], we were unable to detect any evidence of Bd infection and pathology via mouthpart histology from exposed animals at Gosner stage 40 (Supp. Fig. S7). It should be noted that analyses were performed after mouth-part swabbing, and only on one slide per animal, so patchy infections may have been missed [58].

## Discussion

We investigated the immune response of metamorphosing *Mixophyes fleayi* to Bd exposure by comparing immune cell enrichment in the blood and liver of control and exposed animals throughout metamorphosis (Gosner stages 40, 42 and 45). At Gosner stage 45, the majority of exposed animals developed clinical signs (*n* = 21/29, exposed-moribund) and were humanely euthanised, while a subgroup of animals had apparently cleared their Bd infections by the end of the experiment (*n* = 8, exposed-cleared). The histological presence of Bd infection and the associated skin pathology was only identified in moribund animals during the final stages of metamorphosis (Gosner stage 45; Fig. 7). We identified Bd in skin histological sections (Fig. 7) via the presence of full and empty Bd thalli and associated tissue damage (loose adherence of stratum corneum, hyperkeratosis [thickened outer layer] and hyperplasia [increased cell production] [55]). The heightened sensitivity of quantitative molecular methods (qPCR) in comparison to histological detection may explain this discrepancy, as patchy or low-load infections may have been missed [58].

Despite being a primary site for immune cell differentiation [23], spleen tissues were not sampled for these analyses, as the small size of the amphibian spleen necessitated prioritisation for transcriptomics analyses (*unpublished data*). Additional histological analyses including the spleen of metamorphic animals would enhance our understanding of the amphibian immune response during these critical stages. It should also be noted that calculations based on the relative numbers of leukocytes from smears are complicated. At metamorphosis, the removal of larval erythrocytes for replacement by adult cells may lead to leukocyte counts appearing elevated in metamorphosing animals.

We found that the circulating blood and liver leukocyte counts of negative control animals did not significantly differ between Gosner stages (Supp. Fig. S12, S16). Previous studies, however, identified variation in circulating blood leukocyte abundance throughout amphibian development (Gosner stages 26–46) [43]. This discrepancy may result from the comparably small range of developmental stages covered in the present study (Gosner stages 40, 42 and 45), whereas the inclusion of cell counts during the early larval and post-metamorphic stages could potentially reveal more significant differences. We did, however, observe increases in melanomacrophage aggregate abundance and density in control animal liver tissues sampled at Gosner stage 45 (Fig. 6) compared to the earlier sample stages (Supp. Fig. S16, S18). This is consistent with previous studies and is related to macrophage progression and programmed cell death in muscle [59] and liver [31] tissues of metamorphosing amphibians. Melanomacrophages are formed in the liver during the final stages of metamorphosis to assist in the widespread clearance of cells via phagocytosis [60]. Moreover, the elevated melanomacrophage counts observed in livers at Gosner stage 45 may result from macrophage consumption of larval red blood cells via apoptosis. This highlights the extensive reorganisation occurring across metamorphic climax, which has the potential to reduce the immune capacity of hosts undergoing immune-associated restructuring.

### The impact of Bd exposure was minimal prior to Gosner stage 45

We did not identify immune cell infiltration in skin sections sampled at any Gosner stage, including in moribund animals at Gosner stage 45. Leukocyte infiltration in the skin of Bd-infected amphibians is often mild [1, 61], possibly as a result of Bd-induced localised immune suppression within the skin preventing the recruitment of inflammatory cells to the site of infection (e.g. Bd-secreted immunomodulatory metabolites [39, 62, 63]). We found that the impact of Bd exposure was minimal at Gosner stages 40 and 42 (Figs. 2 and 6), with counts of central blood leukocytes (Supp. Table S4-5) and liver immune cells (Supp. Table S12-13) largely consistent between control and exposed groups. This contradicts the variation in control and Bd-exposed tadpole circulating blood leukocyte populations demonstrated previously [42]. The apparent absence of an overt inflammatory response at Gosner stages 40 and 42 may be influenced by the restricted infection burdens identified in Bd-infected tadpoles (infection plateau [12]) and lack of detectable infection at Gosner stage 42 (Supp. Fig. S1) inadequately activating host immune defences. The intracellular nature of Bd infection may be enabling pathogenic evasion of the ordinarily competent host recognition mechanisms. Alternatively, Bd may be actively suppressing host immune defences during the earlier stages of Bd infection [64], as demonstrated by the decreased white blood cell frequencies observed in Bd-infected common green (*Litoria caerulea*) and white-lipped (*Litoria infrafrenata*) tree frogs [38].

Clinical Bd infection was only detected in Bd-exposed animals sampled at Gosner stage 45. A number of factors likely contributed to the onset of clinical signs in *M. fleayi* at this time, such as the dramatic expansion in Bd load following temporary infection clearance at Gosner stage 42 [12], combined with the time needed post-exposure to reach a required infection threshold [65]. Morphological changes and shifts in tissue tropism, driven by the spread of keratinisation from the mouthparts of larval animals to the full body skin of metamorphs [9], drastically expand Bd infection burdens and associated tissue damage, thereby amplifying host immune responses post-Gosner stage 42.

Bd infection burdens may have peaked and ultimately overwhelmed animals before the completion of metamorphosis as the long development time of *M. fleayi* increases the time between Bd tissue trophic expansion with widespread skin keratinisation (Gosner stage 42) and the completion of metamorphosis, thus reducing the stage at which the pathological impacts of Bd become evident (shifting it to pre-completion of metamorphosis). The average duration of metamorphosis (time between Gosner stage 42 and Gosner stage 45—the point of euthanasia) for *M. fleayi* in the present Bd challenge experiment was 49.5 days for controls (*unpublished data*). Despite not including Gosner stage 46, this is considerably longer than the metamorphic duration of 14 taxonomically diverse frog species presented by Downie et al. [66], which ranged from 2.0 to 7.3 days. Additionally, the final metamorphic stages are characterised by extensive energy requirements associated with metamorphic restructuring [19]. The trade-off in resource allocation required following pathogenic invasion and initiation of the immune response is likely complicated by the high energy expenditure of metamorphic restructuring. It should be noted that the findings of the present study could result from the adaptation or plasticity mechanisms of *M. fleayi* specifically, thereby reducing the applicability of our results to other anuran species. Moreover, the *M. fleayi* included in the present study were all hatched from the same egg clutch. This may mean that the genetic diversity of this sample group was lower than if multiple different clutches were examined simultaneously. We thus recommend future studies incorporate pro-metamorphic (Gosner stages 36–41), climax (Gosner stages 42–46) and post-metamorphic stages into analyses to explore host responses within and between a variety of populations and species with diverse ontogeny and disease susceptibility.

### Elevated inflammatory response in moribund animals

Blood leukocyte abundance varied significantly between infection outcome groups (control, exposed-cleared and exposed-moribund) at Gosner stage 45. Both monocytes and neutrophils were significantly elevated in animals sampled at the onset of morbidity (compared to control and cleared groups; Fig. 2). Bd-induced elevation in neutrophil abundance has been demonstrated previously [42, 67–69], despite some inconsistencies within the literature [70, 71]. The mounting inflammatory response could signify impairment to the inflammatory cascade’s regulatory feedback loops artificially amplifying immune expression [8] and ultimately triggering immunopathology [64]. Moreover, white blood cell counts were elevated in moribund animal livers at Gosner stage 45 (Fig. 6), supporting the relationship between Bd exposure and induced leukocyte infiltration through the hepatic parenchyma identified previously [37]. The increase of white blood cells in the liver could represent metabolic stress stimulating a positive feedback cycle of chronic inflammation [72]. Sustained immune response to Bd exposure can negatively impact amphibian survival [73]. The significant relationship between leukocyte abundance and Bd infection load demonstrated in monocytes and neutrophils (Supp. Fig. S14, Supp. Table S8) suggests that the inflammatory response is load-dependent. However, as the present study did not test for immunopathology specifically, the possible role of the inflammatory response in the onset of clinical signs cannot be concluded with any level of certainty.

The heightened neutrophil-to-lymphocyte (N/L) ratio, which characterises the pro-inflammatory state [41], was evidenced in moribund animals at Gosner stage 45 (Supp. Fig. S11). Both lymphocyte and eosinophil counts were reduced in exposed (cleared and moribund) animals compared to same-stage controls (Fig. 2), consistent with Bd-induced lymphocyte suppression. The elevated inflammatory response and reduced lymphocyte abundance are consistent with lymphocyte-suppressive Bd metabolites [39]. Previous research [68] found N/L ratios to be elevated in moribund animals and reduced in surviving animals able to resist Bd infection. Moreover, greater N/L ratios can represent stimulation of the haematopoietic stress response in Bd-infected animals [74, 75], emphasising the dysregulation within moribund animals.

### The role of immune regulation in infection clearance

The observed clearance of Bd infection in a subgroup of animals at Gosner stage 45 (Supp. Fig. S1) was consistent with the comparable monocyte and neutrophil counts between control and exposed-cleared animals at the same stage (Figs. 1, 2), and could indicate that host blood profiles are being restored to pre-infection levels. In contrast, liver leukocyte abundance was reduced in the exposed-cleared subgroup to levels lower than naïve controls (Fig. 6). This is consistent with Young et al. [38], who found that Bd-exposed, white-lipped tree frogs (*Litoria infrafrenata*) capable of self-clearance had lower liver leukocyte counts than unexposed animals. There is the potential for long-term immunological impacts of Bd infection clearance [73], as hosts may be redirecting resources to processes that better support animal recovery [38]. However, in the absence of long-term post-metamorphic monitoring, we cannot confirm this hypothesis.

The reduction in infiltrating leukocytes in the livers of animals that successfully cleared Bd infections could either be a consequence of prior cellular removal [40] or the effective limitation of inflammation via initiation of host feedback mechanisms [76]. We previously demonstrated that animals capable of infection clearance at Gosner stage 45 did not have detectable infections at Gosner stage 42, but had apparently re-gained high-level infections before secondary clearance at Gosner stage 45 [12]. Despite the lack of immune cell data during these intermediary stages, we did have one animal sampled at Gosner stage 45 that had detectable Bd infection but was not displaying clinical signs at the time of sampling (B25, Supp. Fig. S1−2). The reduction in B25 infection loads (to levels lower than moribund or larval animals; Supp. Fig. S1) could suggest a progression towards clearance, rather than morbidity, in this animal. This was supported by the Bd load trajectory of B25 over time and throughout development, which shows a peak in infection loads (to clinical infection levels) at Gosner stage 45 followed by sequential losses over subsequent weeks (Supp. Fig. S2).

The blood leukocyte abundance of B25 generally matched the exposed-cleared infection group (Supp. Fig. S10, S13), with lower monocytes and neutrophils than moribund animals. The substantial elevation in circulating blood lymphocytes in B25 (compared to all other sample groups) could indicate the initiation of an adaptive immune response via effective pathogen recognition (antigen-presenting cells), selection, clonal amplification and differentiation of lymphocytes [40]. This is in stark contrast to the elevated N/L ratios evidenced in moribund animals (Supp. Fig. S11) and suggests that the cleared subgroup was able to overcome Bd-induced lymphocyte suppression. The liver leukocyte abundance of B25 also mirrors the exposed-cleared group (lower counts than moribund or controls; Supp. Fig. S17), indicating hepatic immune cell consumption. Despite not being statistically robust, these results provide important insights into the potential mechanisms (adaptive immunity [77]) responsible for eventual infection clearance in some metamorphic *M. fleayi*. Targeted gene expression analyses (e.g. immunomodulatory cytokines) would allow us to better establish the drivers of the varied immune responses observed across amphibian development and the role they play in infection clearance.

### Conclusions and future directions

Amphibian metamorphosis is characterised by dramatic morphological remodelling, immune restructuring, high energy utilisation and a shift in the tissue tropism of Bd. Each of these features of *Mixophyes fleayi* metamorphosis, particularly post-Gosner stage 42 (the onset of climax), could be contributing to the observed severe pathological outcomes caused by chytridiomycosis at this life stage. We did not observe any significant impacts of Bd exposure until the final stages of metamorphosis (Gosner stage 45), with blood and liver leukocyte counts consistent between control and exposed groups at Gosner stages 40 and 42. At Gosner stage 45, the elevated immune response identified in moribund animal blood and liver samples is reminiscent of the amplified inflammatory response driving immunopathology in clinically infected amphibians. This heightened immune expression could either be a direct consequence of heavy Bd infection burdens, associated epithelial damage and potential secondary infections (although not observed) or stimulation of the haematopoietic stress response. The reduced liver leukocyte abundance (to levels lower than naïve controls) observed in the exposed-cleared subgroup may be a consequence of prior cellular consumption or effective immune regulation. We tentatively present evidence for the initiation of adaptive immune components in one animal that appeared to be in the intermediary stages of infection clearance. Although not statistically robust, this presents a possible mechanism by which animals were able to clear Bd in the final stages of metamorphosis. We thus recommend future studies use targeted gene expression analyses (e.g. immunomodulatory cytokines) to explore the mechanisms responsible for the possible immunopathology and infection clearance observed at metamorphic climax.

## Supplementary Information

Below is the link to the electronic supplementary material.

Supplementary file1 (DOCX 84659 KB)

## Data Availability

Data is provided within the manuscript or supplementary information files.
