# Supplementary material for: Amphibian cellular immune response to chytridiomycosis at metamorphic climax
Source: Immunol Res. 2025 Jan 30;73(1):44. doi: 10.1007/s12026-025-09599-5 (PMC11782352; doi:10.1007/s12026-025-09599-5)
Supplement: Supplementary file 1 — Supplementary file1 (DOCX 84659 KB) [file 12026_2025_9599_MOESM1_ESM.docx]

Supplementary Information - Immunologic Research

**Amphibian cellular immune response (blood, skin and liver) to chytridiomycosis at metamorphic climax**

Josephine E. Humphries ^a,b^*, Allan Hicks ^c^, Chantal Lanctôt ^a,d^, Hamish McCallum ^b^, David Newell ^e^, & Laura F. Grogan ^a,b,f^

^a^ School of Environment and Science, Griffith University, Southport, Queensland 4222, Australia.

^b^ Centre for Planetary Health and Food Security, Griffith University, Southport, Queensland 4222, Australia.

^c^ School of Pharmacy and Medical Sciences, Griffith University, Southport, Queensland 4222, Australia

^d^ Australian Rivers Institute, Griffith University, Southport, Queensland 4222, Australia

^e^ Faculty of Science and Engineering, Southern Cross University, Lismore, New South Wales 2480, Australia.

^f^ School of the Environment, University of Queensland, St Lucia, Queensland 4067, Australia.

**^*^Corresponding author:** Josephine E. Humphries.

Address: Centre for Planetary Health and Food Security, Griffith University, Southport, QLD 4222, Australia. Email: [josie.humphries@griffithuni.edu.au](mailto:josie.humphries@griffithuni.edu.au). Phone: +61 447 651 282. ORCID: 0000-0001-9094-3982.

1. Bd exposure experiments


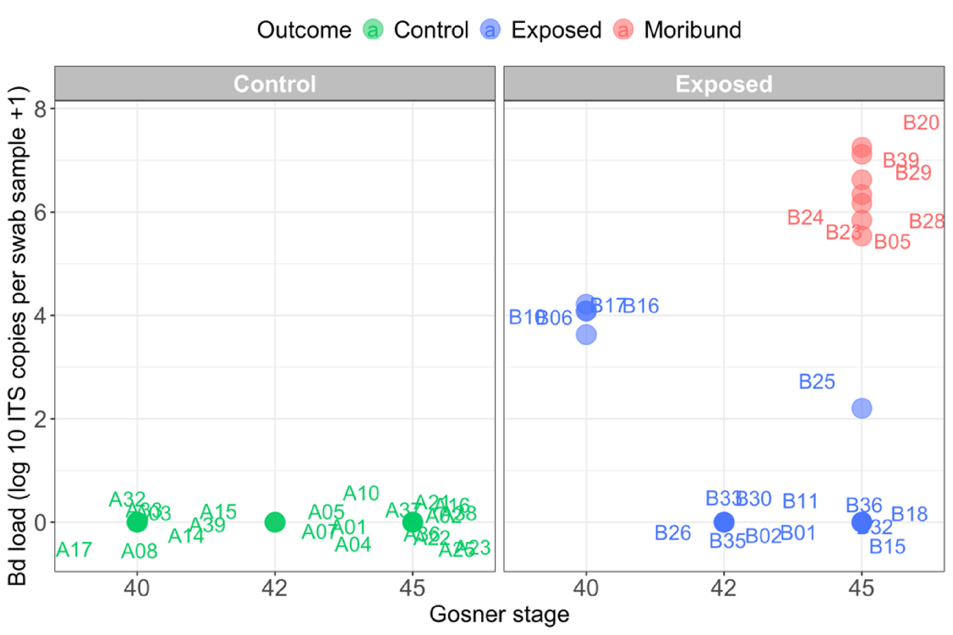


**Fig. S1** Bd load (log 10 ITS copies per swab sample +1) qPCR output from swab samples taken at the point of sampling (euthanasia). Animals were sampled at Gosner stages 40 (6 control, 5 exposed), Gosner stage 42 (6 control, 5 exposed), and Gosner stage 45 (13 control, 29 exposed [8 animals sampled pre-clinically, plus 21 demonstrating overt clinical signs of disease]). Each label represents an animal ID code, coloured by exposure group and infection outcome, and the position of points is jittered horizontally.


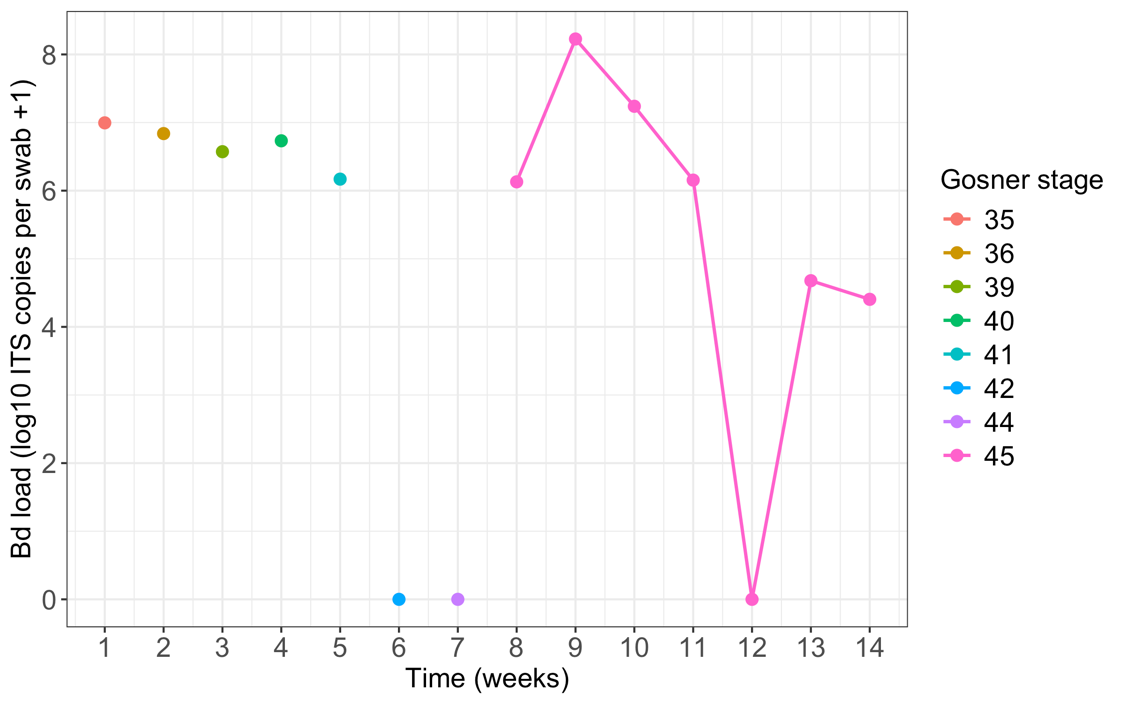


**Fig. S2** Bd load progression of exposed animal (B25) leukocyte proportions at the point of sampling (Gosner stage 45). B25 was an outlier, as it was the only exposed animal that did not develop clinical signs despite still having detectable infections at sampling.

**Table S1** The number of specimen slides included in the statistical analyses of each cell stain; H&E and Toluidine blue for skin and liver histology samples, and Wright’s-stain for blood smears. The variability in sample size numbers is a result of differential specimen quality.

| Exposure | Gosner stage | H&E stain | Toluidine Blue | Wright’s stain |
| --- | --- | --- | --- | --- |
| Control  Exposed | 40  40 | 6  5 | 5  5 | 6  4 |
| Control  Exposed | 42  42 | 6  5 | 5  4 | 4  5 |
| Control  Exposed  Moribund | 45  45  45 | 13  8  21 | 10  5  13 | 12  7  7 |
| Control  Exposed  Total | **All**  **All**  **All** | **25**  **39**  **64** | **20**  **27**  **47** | **22**  **23**  **45** |

1. Sample processing in QuPath

We applied a grid to each specimen (250x250µm) and randomly selected a grid square to perform cell counts. We created a region annotation (Classify 🡪 Training images 🡪 Create region annotation) at a random location on the specimen and aligned the square to the nearest grid space. We created 20 grid squares (field of view; FOV) per specimen (animal). We used automated cell detection to identify cells (Analyse 🡪 Cell detection). Baseline features were set for initial detections (Optical density, pixel size=0.5, background radius=3, median filter radius=0, sigma=1.5, threshold=0.1, background intensity=5, cell expansion=5, minimum area=5) and modified to customise detections for each specimen. Cell identification was based on nuclei optical cell density.

For histological samples, in order to train the cell classifier, we used the brush tool to identify and annotate known cell regions across the specimen. This was repeated until all key cell types had been identified. Training software was then used to classify cell types (Classify 🡪 Object classification 🡪 train object classifier). The validity of initial cell annotations was manually checked, and annotations were adjusted accordingly, repeating this process until cells were reliably classified across the tissue sample. Cell counts were automatically recorded per cell type for each selected FOV (raw data available). This process was then repeated for all 20 selected FOV. This data could also be used to calculate cell density for large aggregates, for which counting alone would not reflect the extent of infiltration. For blood smears, grids were applied and FOV selected in the same manner as for histological analyses (250x250µm). Cell detection was also used to count RBCs, deleting any detected WBCs to enable manual identification and collation of leukocyte counts.


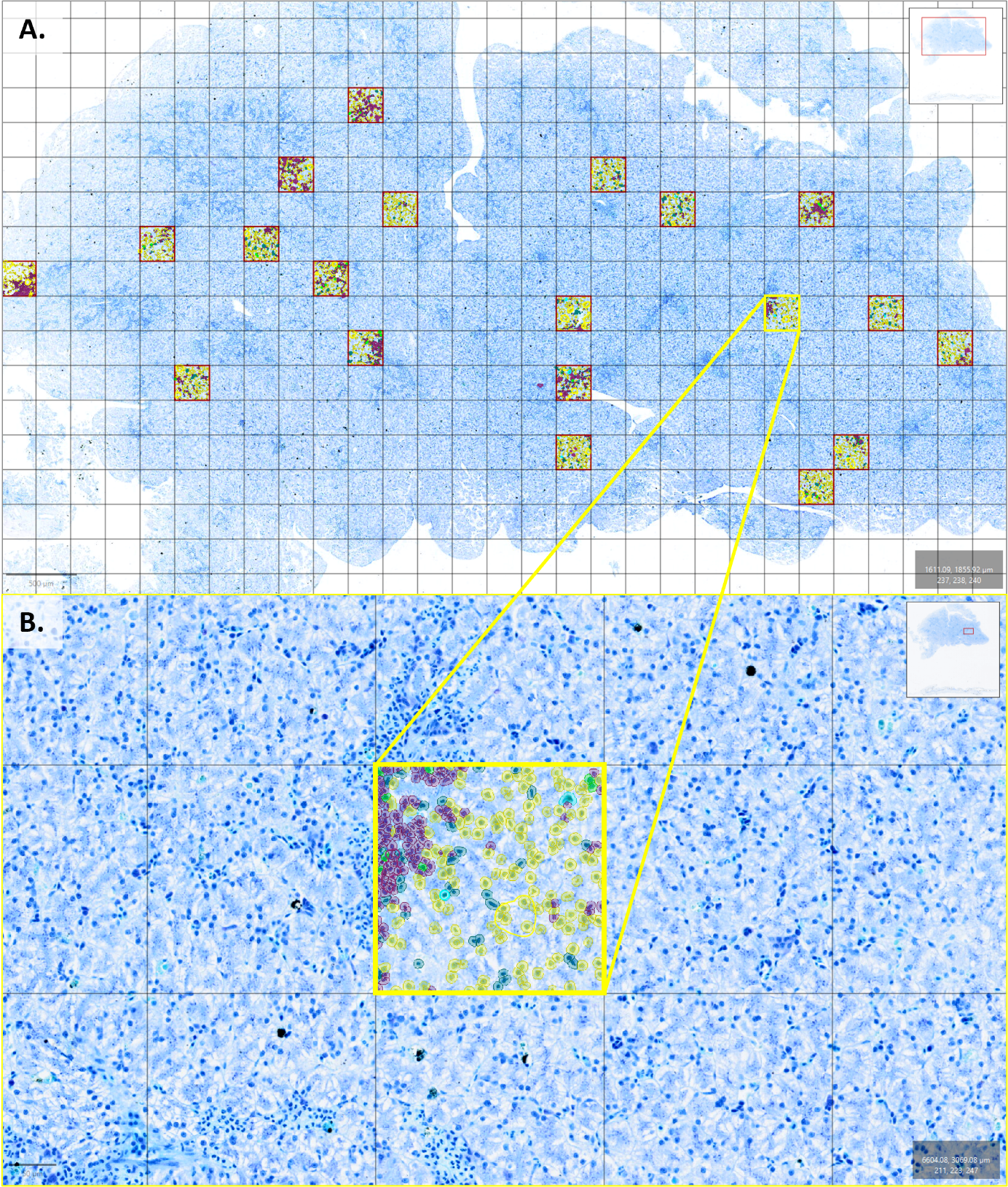


**Fig. S3** Example of the cell detection process for Toluidine Blue stained liver histological sample using QuPath digital pathology and image analysis software (Bankhead et al., 2017). The field of view (FOV) selection process (A), and cell annotation and classification to measure cell counts per FOV (B). Scale bar = 500μm (A) and 50μm (B).


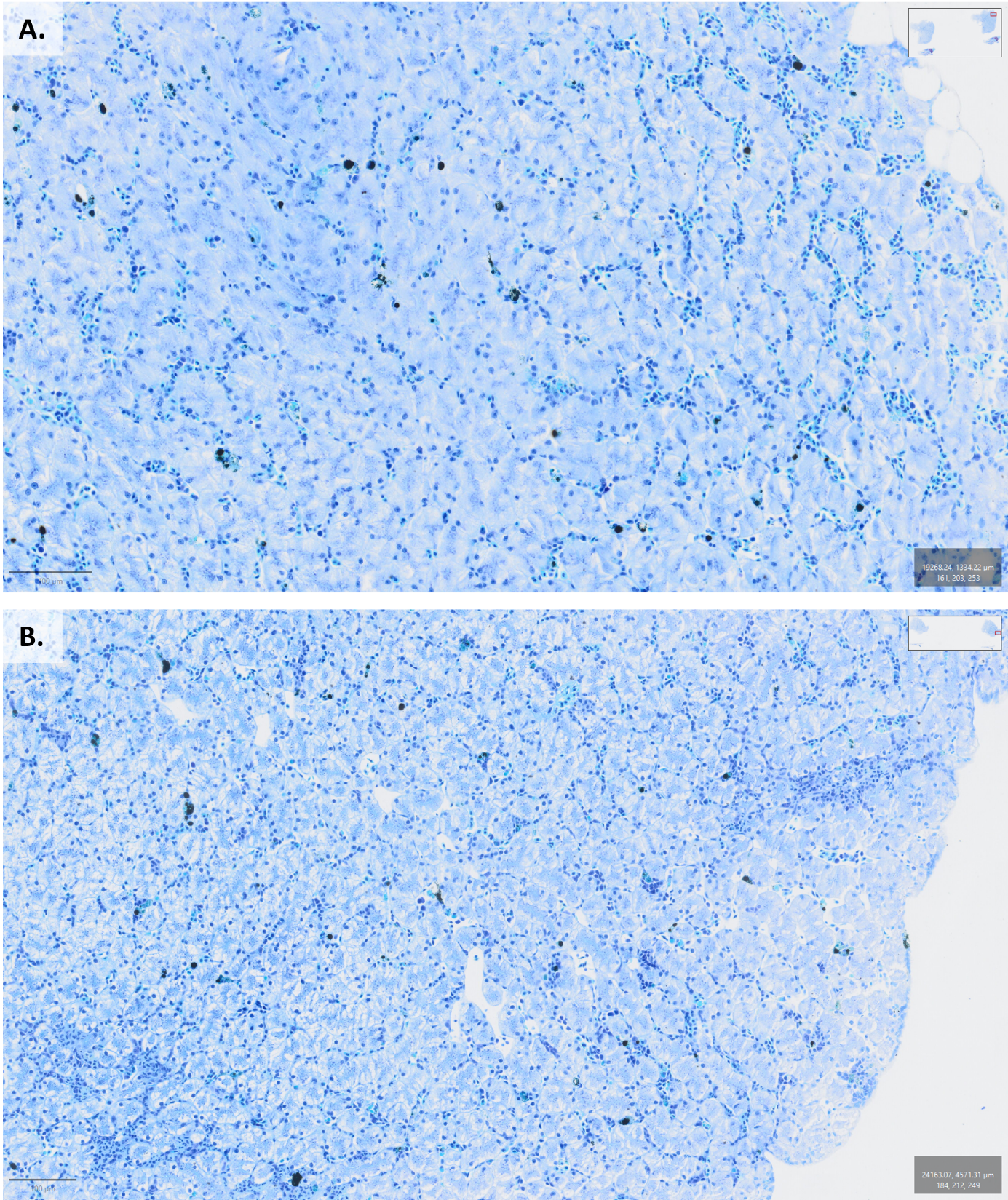


**Fig. S4** Example of a typical liver histological sample (Toluidine Blue stain) of control (A) and exposed (B) animals (*M. fleayi*) at Gosner stage 40. Images are taken from QuPath digital pathology and image analysis software (Bankhead et al., 2017). Scale bar = 100μm.


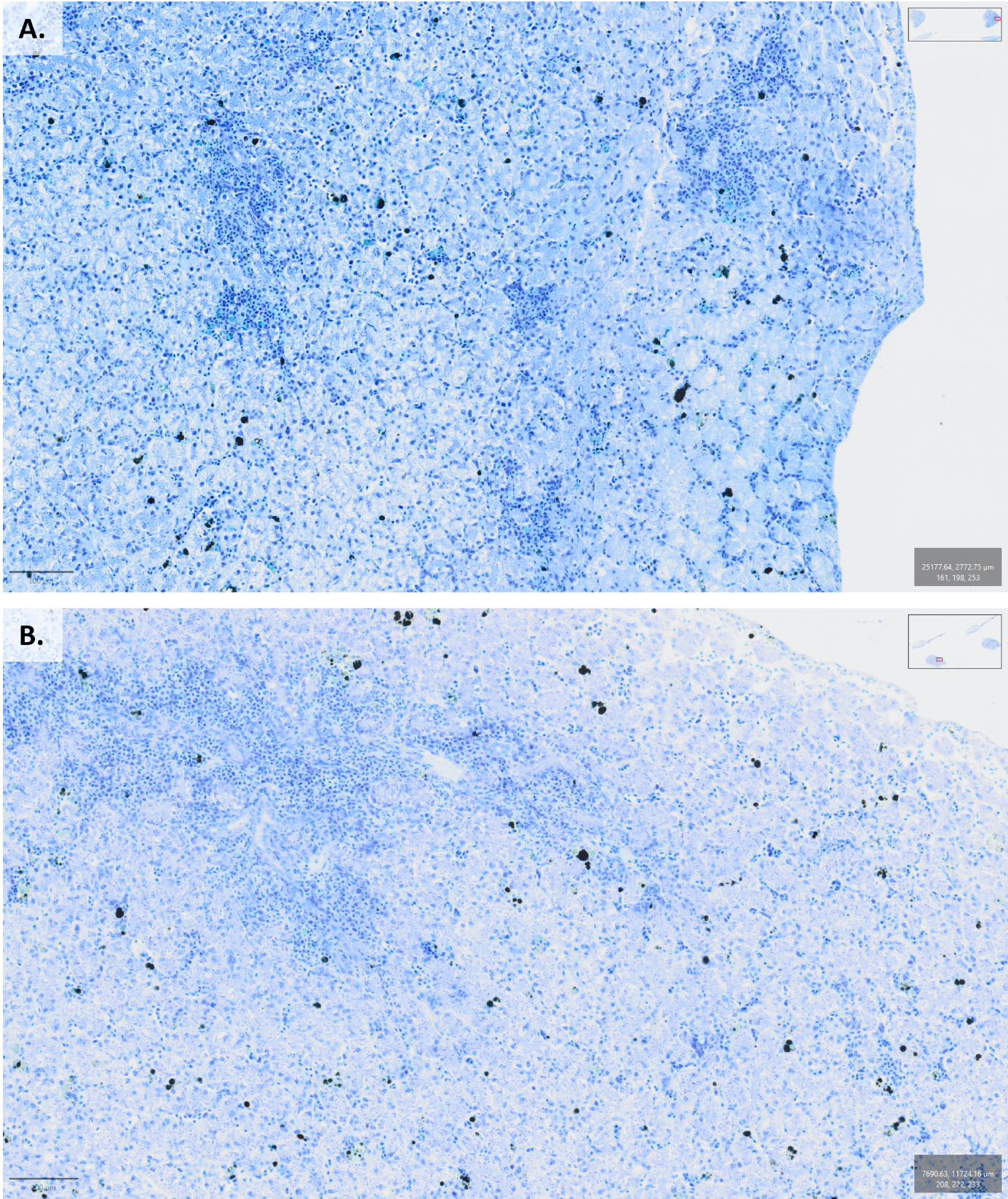


**Fig. S5** Example of a typical liver histological sample (Toluidine Blue stain) of control (A) and exposed (B) animals (*M. fleayi*) at Gosner stage 42. Images are taken from QuPath digital pathology and image analysis software (Bankhead et al., 2017). Scale bar = 100μm.


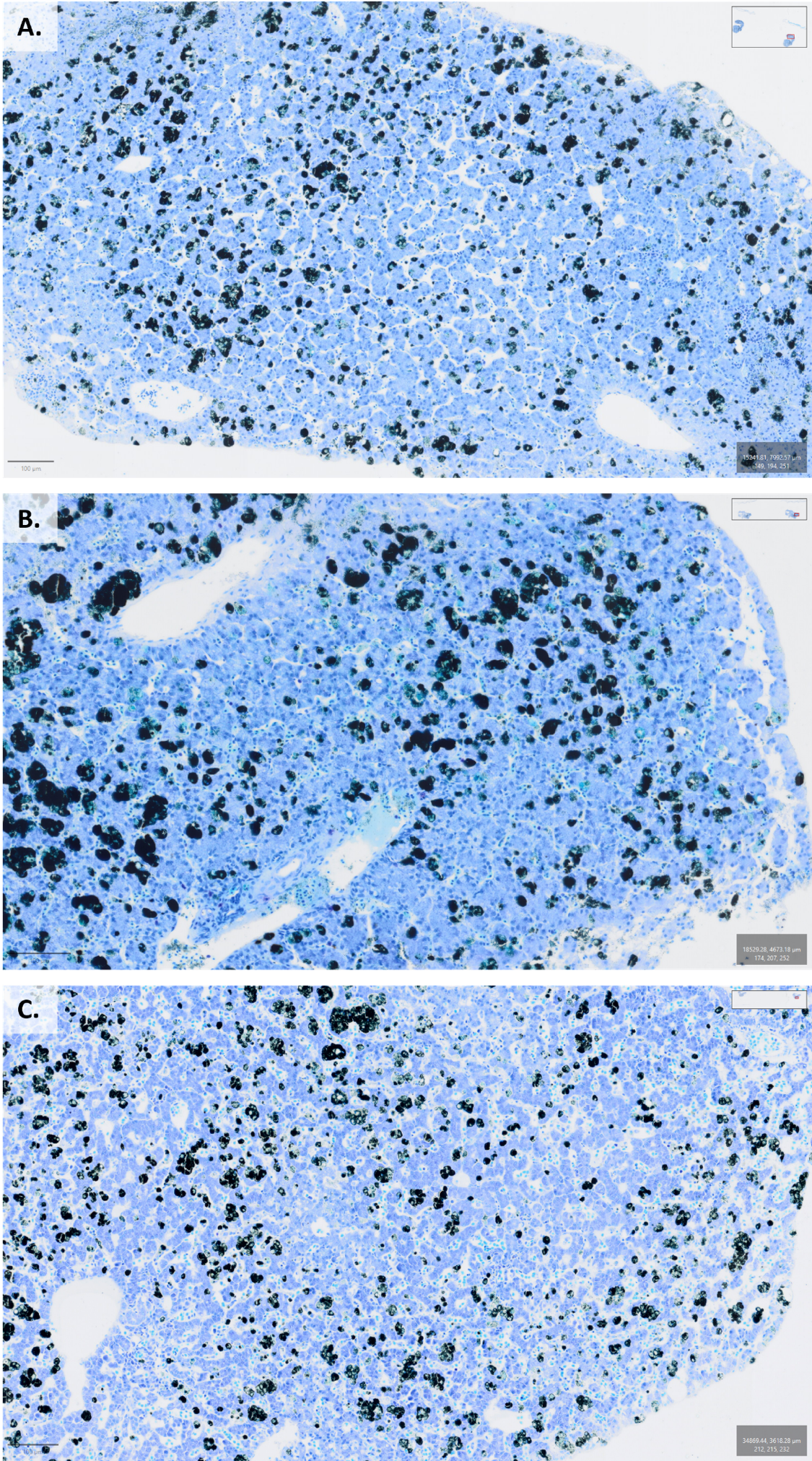


**Fig. S6** Example of a typical liver histological sample (Toluidine Blue stain) of control (A), exposed-cleared (B), and exposed-moribund (C) animals (*M. fleayi*) at Gosner stage 45. Images are taken from QuPath digital pathology and image analysis software (Bankhead et al., 2017). Scale bar = 100μm.


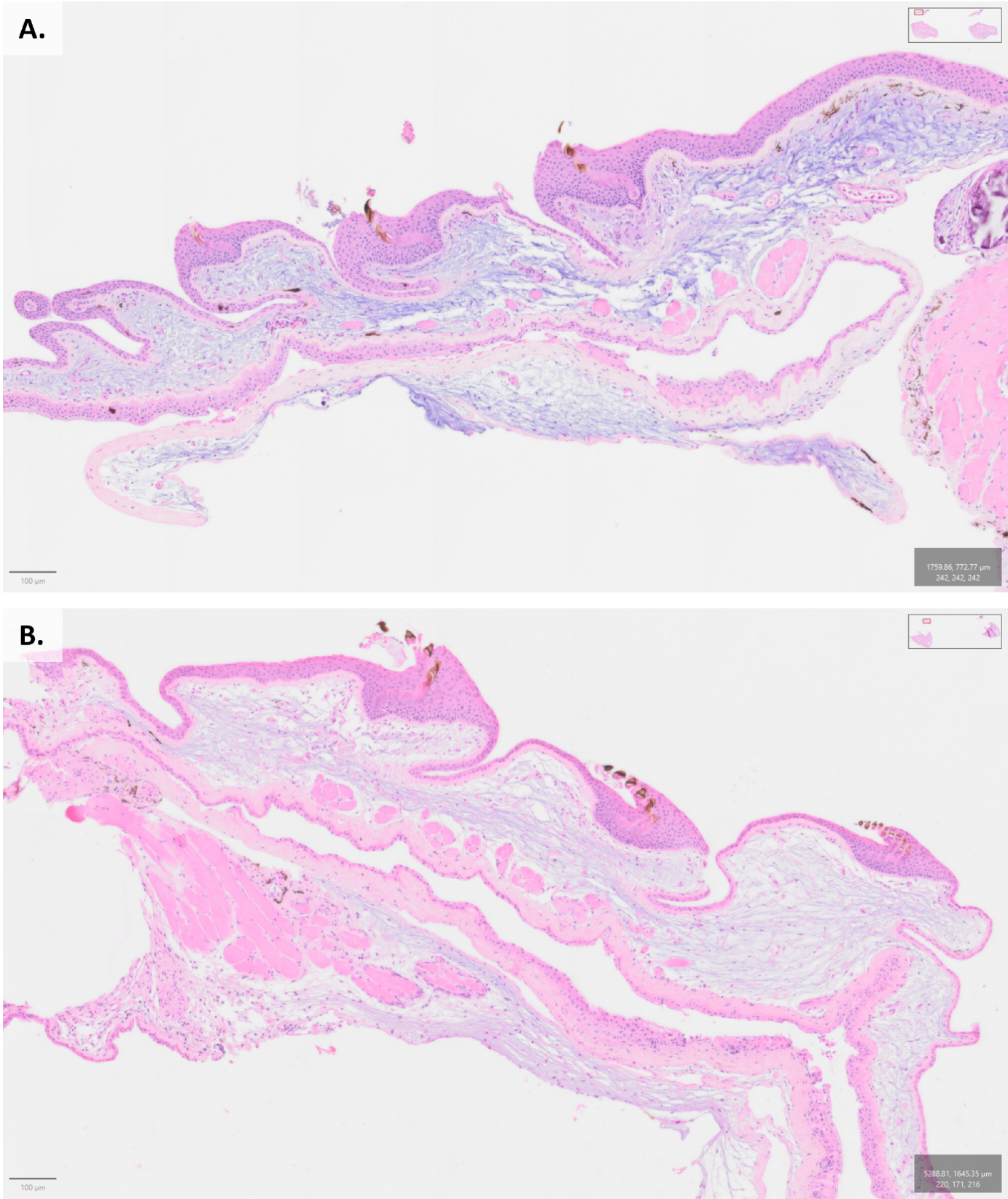


**Fig. S7** Example of a typical skin histological sample (H&E-stained) of control (A) and exposed (B) animals (*M. fleayi*) at Gosner stage 40. Images are taken from QuPath digital pathology and image analysis software (Bankhead et al., 2017). Scale bar = 100μm.


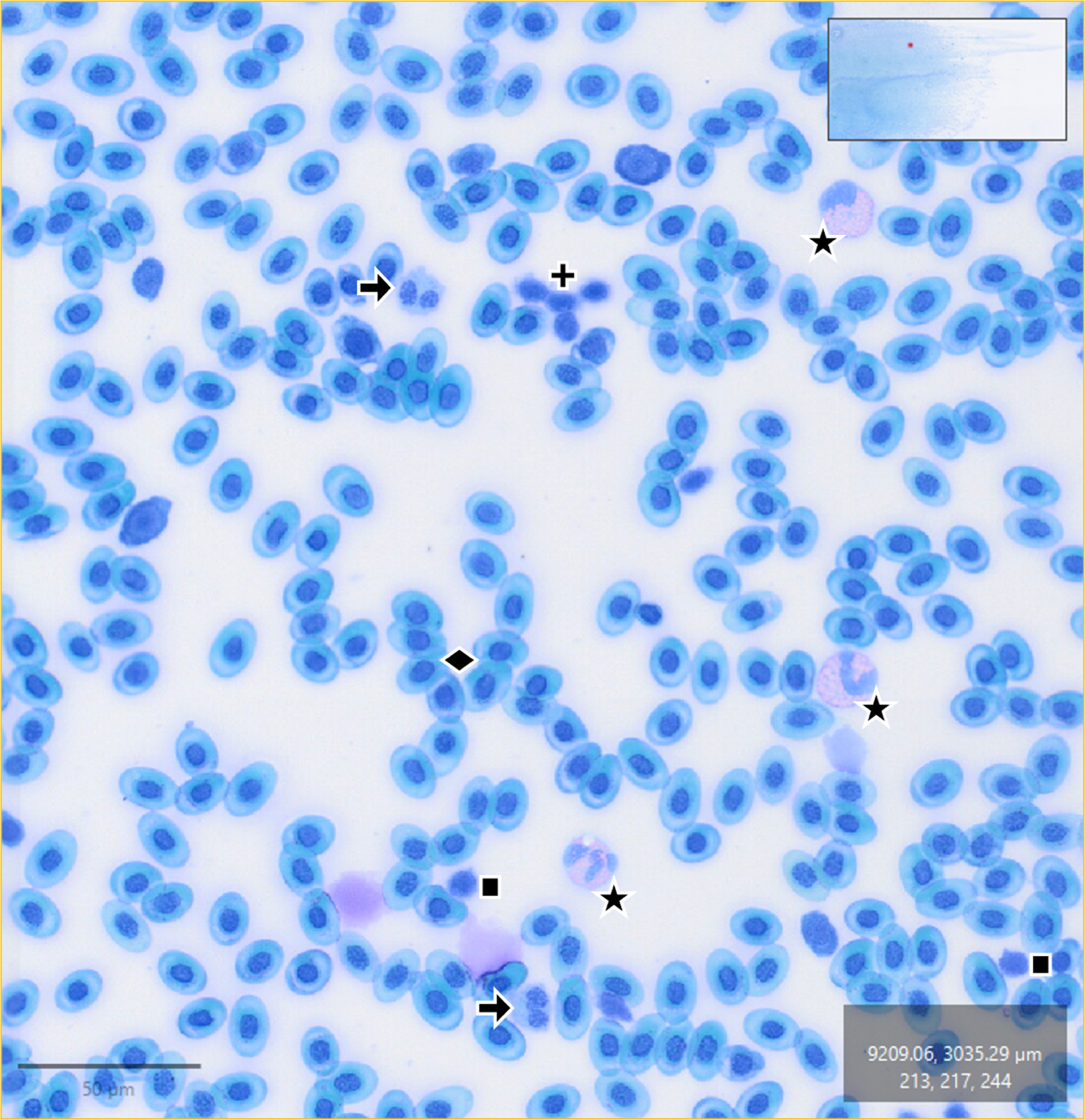


**Fig. S8** Example of blood cell detection in Wright’s-stained blood smears (*M. fleayi*): erythrocytes (diamond), monocytes (triangle), lymphocytes (square), neutrophils (arrow), basophils (X) and eosinophils (star). Images are taken from QuPath digital pathology and image analysis software (Bankhead et al., 2017). Scale bar = 50μm.


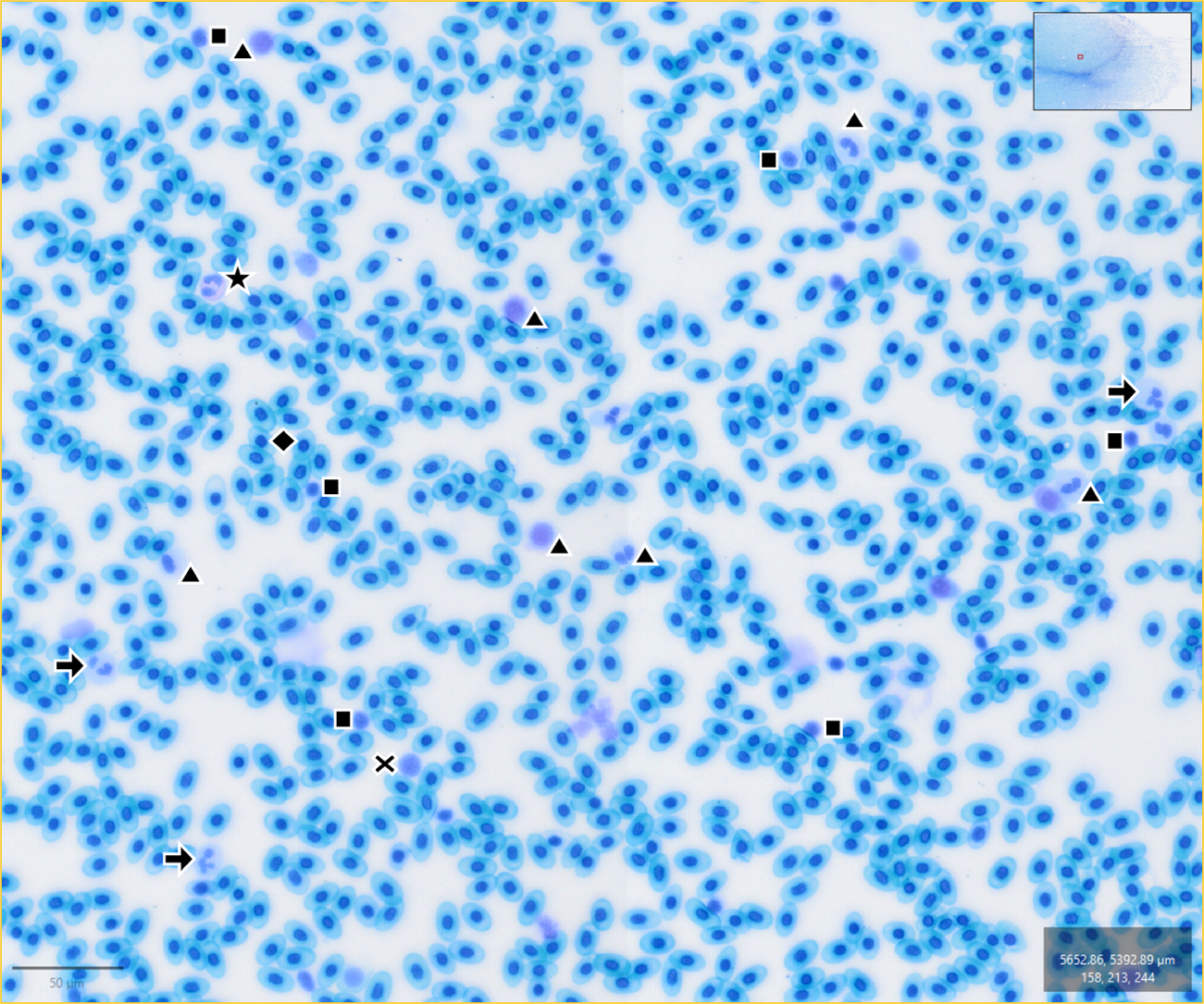


**Fig. S9** Example of blood cell detection in Wright’s-stained blood smears (*M. fleayi*): erythrocytes (diamond), neutrophils (arrow), eosinophils (star), and thrombocytes (cross). Images are taken from QuPath digital pathology and image analysis software (Bankhead et al., 2017). Scale bar = 50μm.

1. Cell counts

**Table S2** Average cell counts for each Gosner stage (40, 42 or 45) and exposure group (control and exposed) performed using Wright’s-stained blood smears.

| Exposure | Stage | N | WBC | RBC |
| --- | --- | --- | --- | --- |
| Control  Exposed | 40  40 | 6  4 | 102.5  73.0 | 2148.2  2529.5 |
| Control  Exposed | 42  42 | 4  5 | 93.8  249.4 | 2452.0  3613.0 |
| Control  Exposed | 45  45 | 12  14 | 146.8  167.9 | 3356.8  4102.9 |
| Average | **All** | **45** | **147.5** | **3260.5** |

- 1. Wright’s-stained blood smears


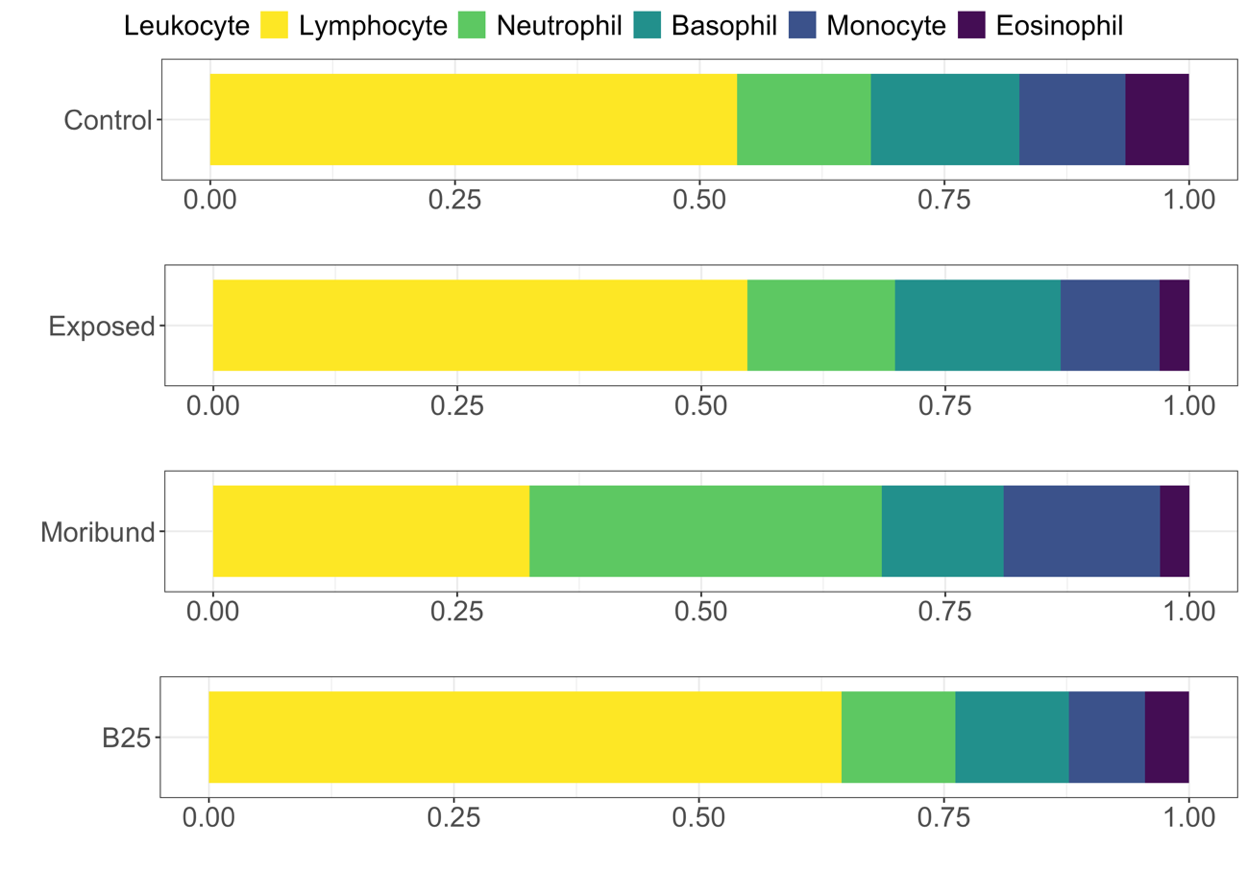


**Fig. S10** Proportional blood leukocyte counts of control, exposed and moribund animals (*M. fleayi)* sampled at Gosner stage 45, in addition to an individual exposed animal (B25) leukocyte proportions at the point of sampling (Gosner stage 45). B25 was an outlier, as it was the only exposed animal that did not develop clinical signs despite still having detectable infections at sampling. Leukocyte counts were performed using blood smears stained with Wright’s stain and values were calculated as a proportion of the total leukocyte counts.


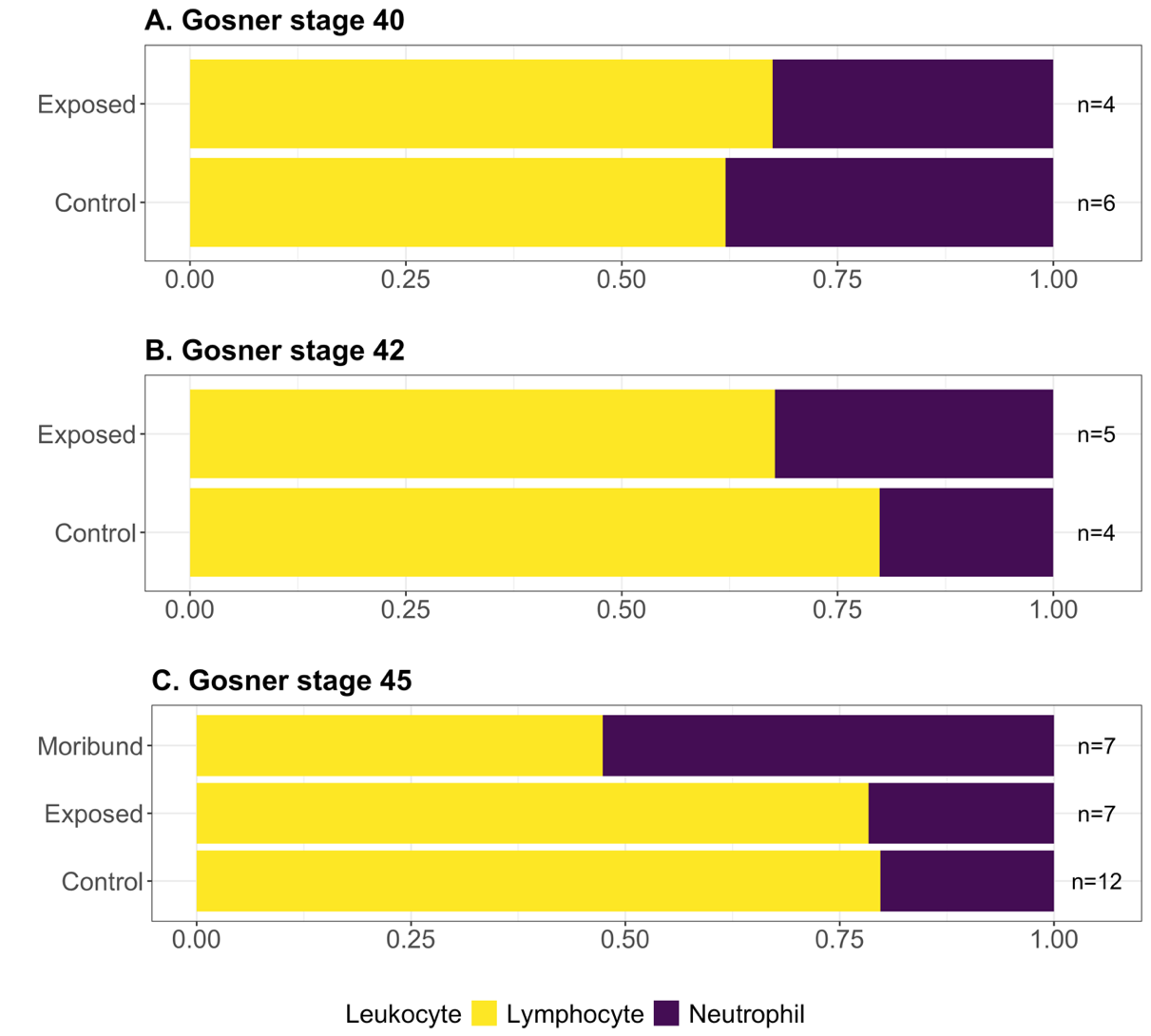


**Fig. S11** Proportional neutrophil to lymphocyte (N/L) counts for each Gosner stages 40 (A), 42 (B) and 45 (C), and sample group (control, exposed or moribund animals). Leukocyte counts were performed using blood smears stained with Wright’s stain and values were calculated as a proportion of the total leukocyte counts.


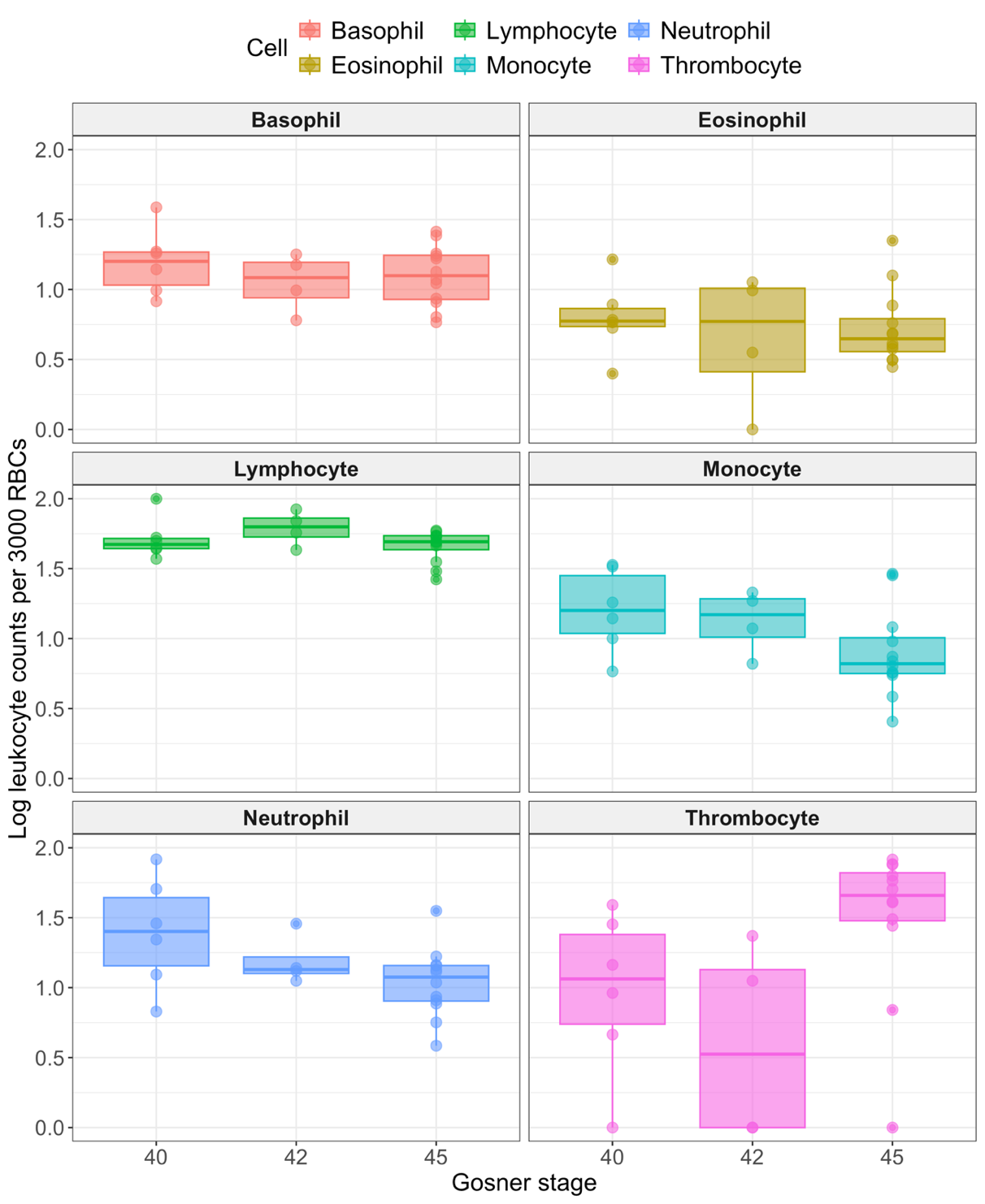


**Fig. S12** Log-transformed cell counts for basophils (A), eosinophils (B), lymphocytes (C), monocytes (D), neutrophils (E), and thrombocytes (F), from control animals throughout development (Gosner stages 40, 42 and 45). Cell counts were performed using 20 randomly selected FOV (250x250µm) of blood smears stained with Wright’s stain and values. Boxplots show the interquartile range (column), median (horizontal line), and minimum and maximum values excluding outliers (whiskers).


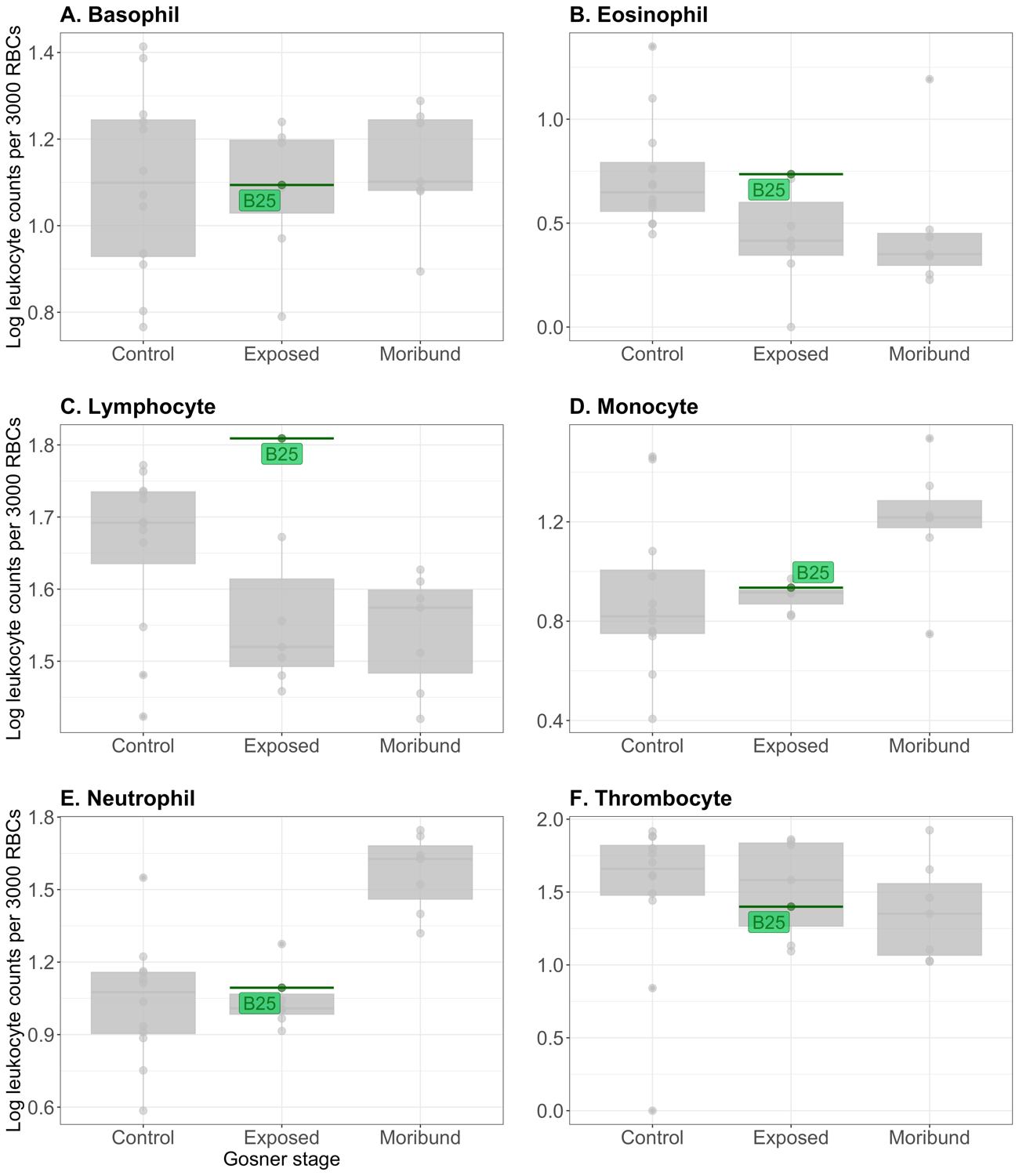


**Fig. S13** Log-transformed cell counts for animals sampled at Gosner stage 45. Exposed animal, B25, is highlighted as an outlier, as it was the only exposed animal that did not develop clinical signs despite still having detectable infections at sampling. Basophils (A), eosinophils (B), lymphocytes (C), monocytes (D), neutrophils (E), and thrombocytes (F), from 20 randomly selected FOV (250x250µm) per specimen (5mm^2^). Cell counts were performed using blood smears stained with Wright’s stain and values. Boxplots show the interquartile range (column), median (horizontal line), and minimum and maximum values excluding outliers (whiskers).


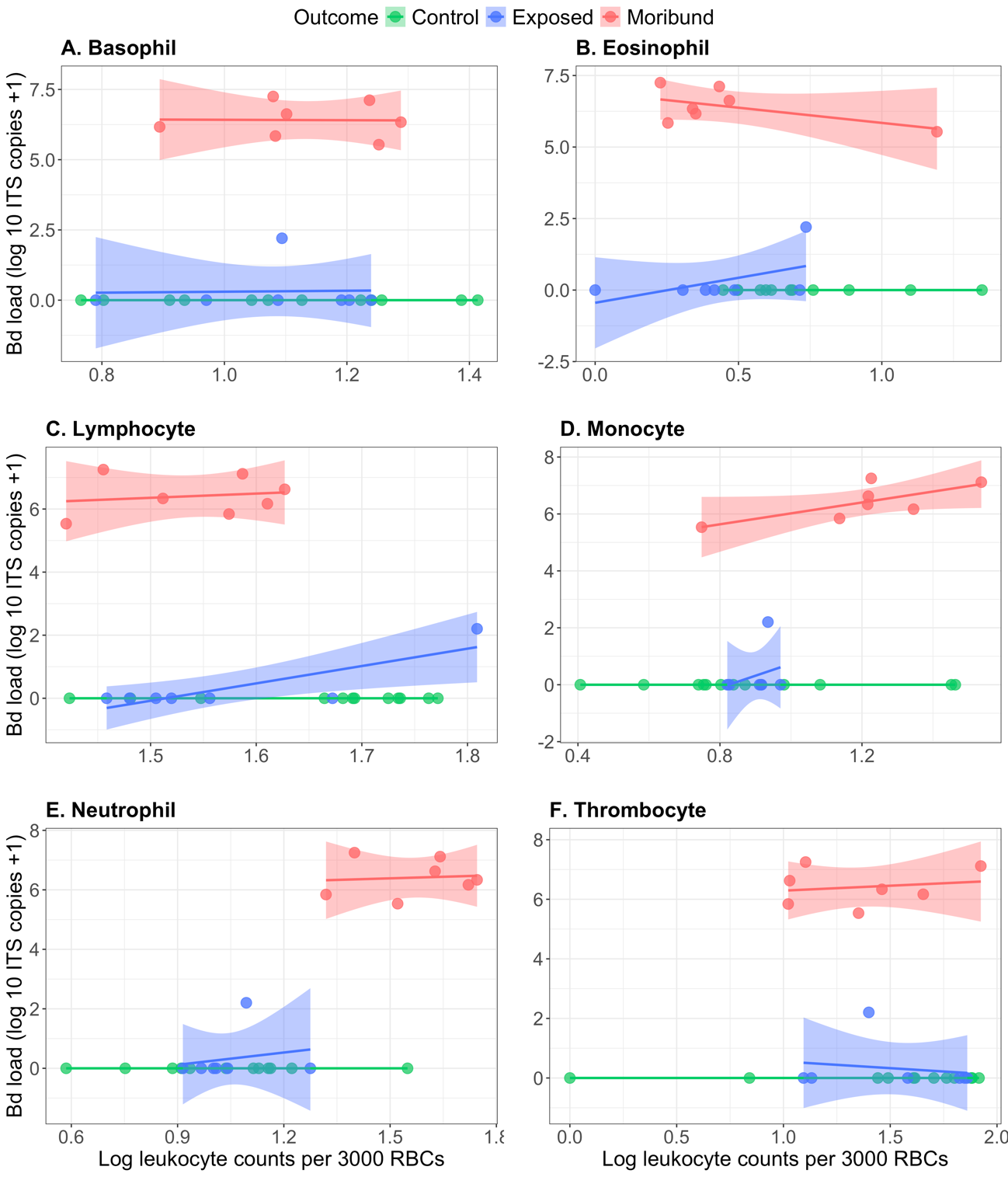


**Fig. S14** The relationship between Bd load (log 10 ITS copies per swab sample +1) qPCR output from swab samples taken at the point of sampling (euthanasia) and log-transformed cell counts for basophils (A), eosinophils (B), lymphocytes (C), monocytes (D), neutrophils (E), and thrombocytes (F), from 20 randomly selected FOV (250x250µm) per specimen (5mm^2^).

- 1. Toluidine Blue stained liver histology samples

**
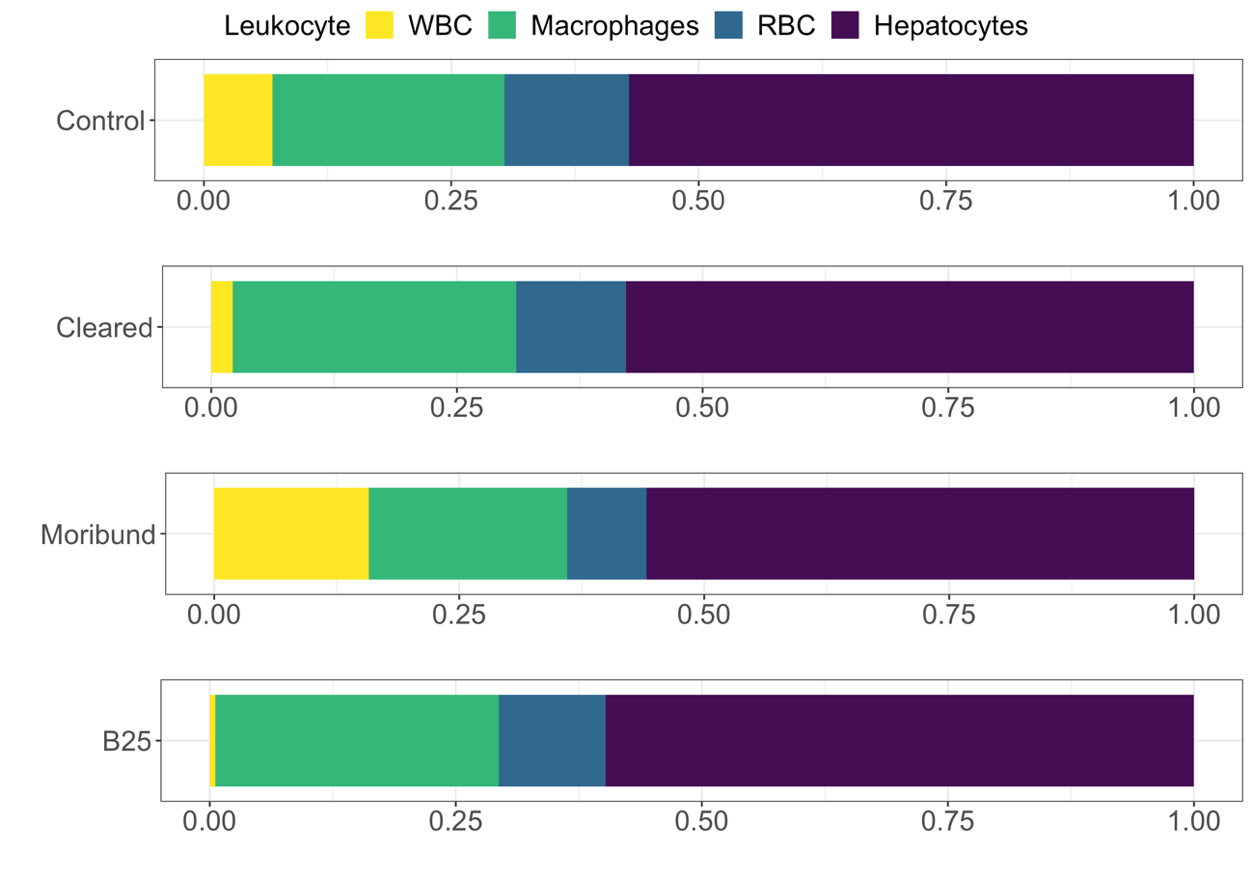
**

**Fig. S15** Proportional counts of white blood cells (WBC), hepatocytes, and melanomacrophages in the liver between Gosner stages 40 (A), 42 (B) and 45 (C), and sample group (control, exposed or moribund animals). Cell counts were performed using histological liver samples stained with Toluidine Blue and values were calculated as a proportion of the total cell counts.


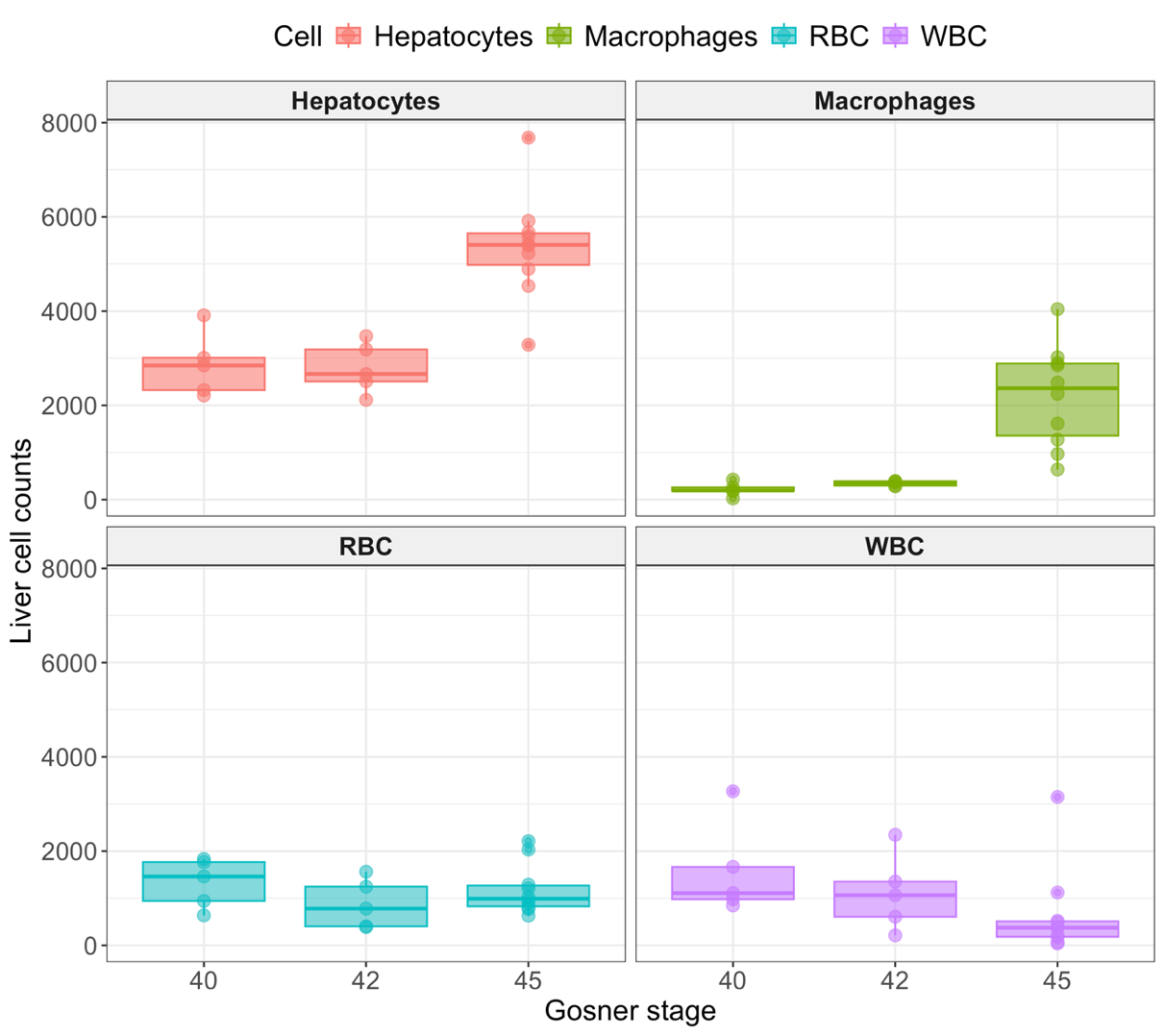


**Fig. S16** Cell counts of white blood cells (A), melanomacrophages (B), red blood cells (C), and hepatocytes (D) from control animals throughout development (Gosner stages 40, 42 and 45). Cell counts were performed using 20 randomly selected FOV (250x250µm) of histological liver samples stained with Toluidine Blue. Boxplots show the interquartile range (column), median (horizontal line), and minimum and maximum values excluding outliers (whiskers).


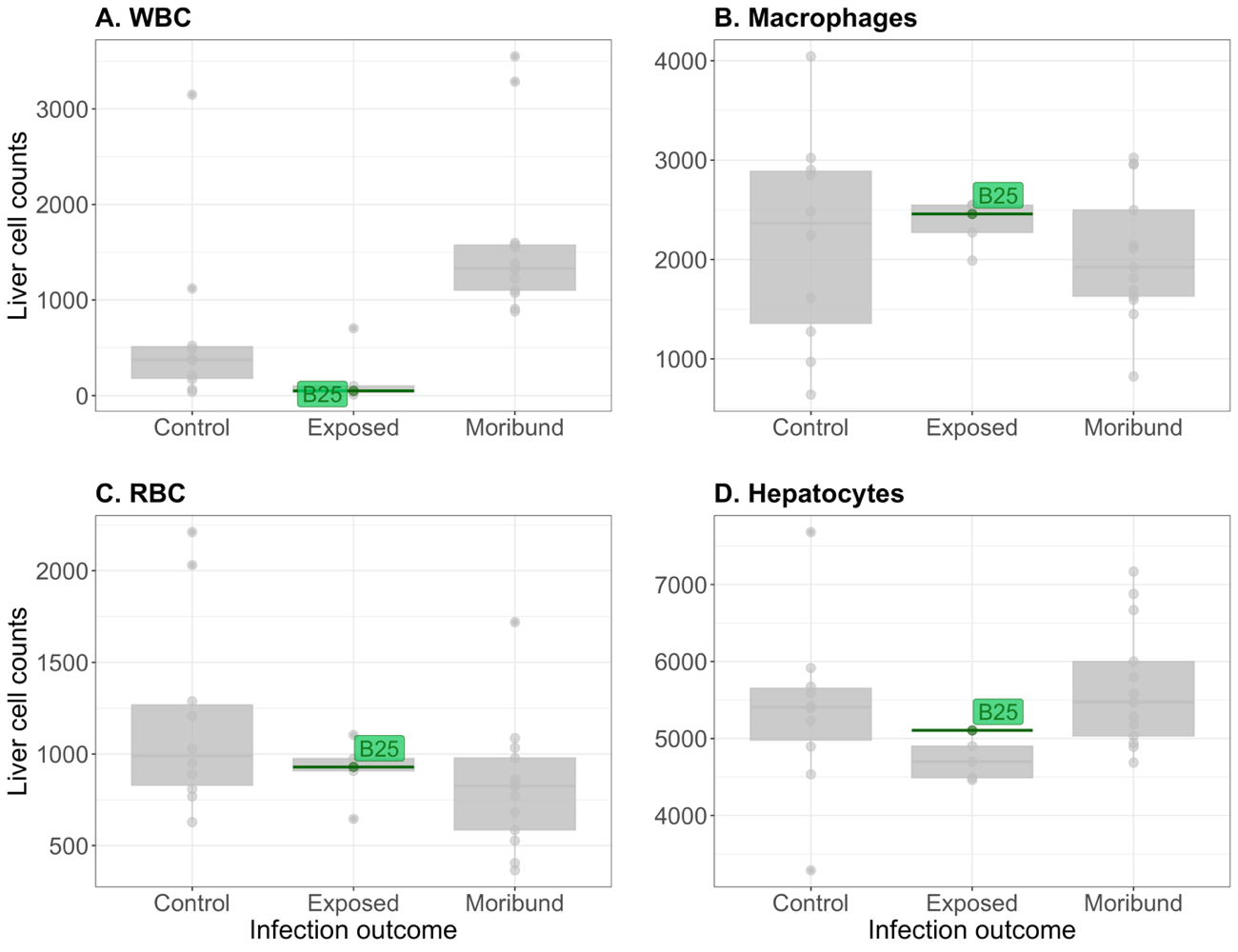


**Fig. S17** Cell counts of (A) white blood cells (WBC), (B) melanomacrophages, (C) red blood cells (RBC), and (D) hepatocytes of animals sampled at Gosner stage 45. Exposed animal, B25, is highlighted as an outlier, as it was the only exposed animal that did not develop clinical signs despite still having detectable infections at sampling. Cell counts were performed on 20 randomly selected FOV (250x250µm) of histological liver samples stained with Toluidine Blue. Boxplots show the interquartile range (column), median (horizontal line), and minimum and maximum values excluding outliers (whiskers).


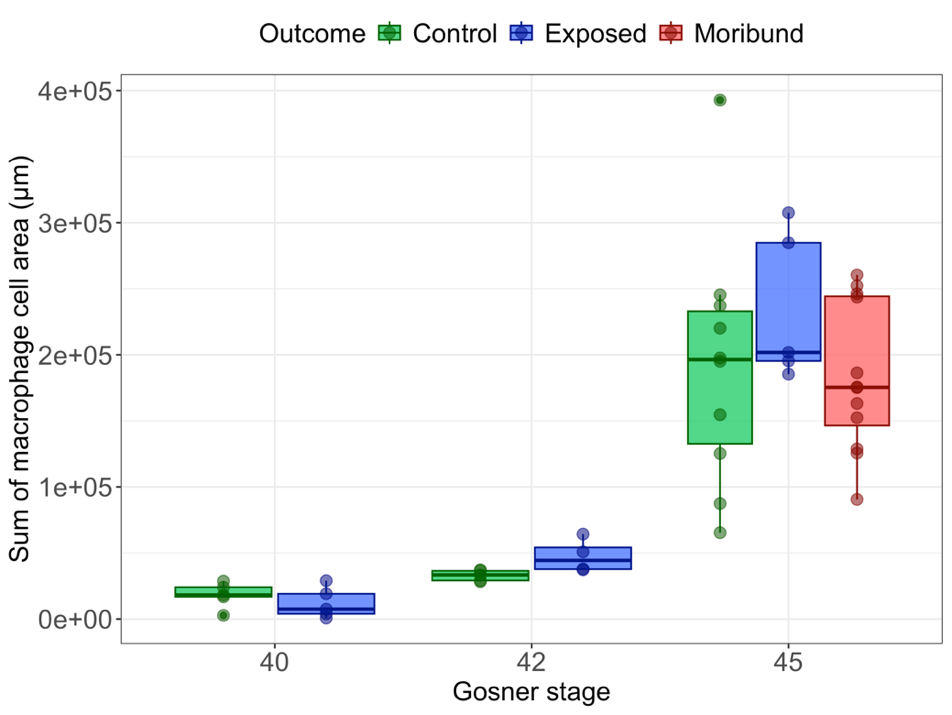


**Fig. S18** Log-transformed sum of pigmented areas of melanomacrophage aggregates (density) from 20 randomly selected FOV (250x250µm) per specimen (5mm^2^). Density counts are coloured by sample group (control, exposed or moribund animals) throughout metamorphic development (Gosner stages 40, 42 and 45). Densities were calculated using histological liver samples stained with Toluidine Blue. Boxplots show the interquartile range (column), median (horizontal line), minimum and maximum values excluding outliers (whiskers), and box widths proportional to the square-root of n.

1. Statistical analyses
   1. Wright’s-stained blood smears

**Table S3** Overall output for one-way multivariate analysis of variance (MANOVA) for Wright’s-stained blood smears of control animals. Proportions of each leukocyte were calculated per 3000 RBCs and numbers were log-transformed (log_10_[count+1]) to approximate normal distributions. The log-transformed proportion of each leukocyte type were the (six) response variables, and Gosner stage (40, 42 and 45) was the predictor variable.

| Variable | Df | Pillai’s Trace | Approx. F-statistic | Num Df | Den Df | P-value |
| --- | --- | --- | --- | --- | --- | --- |
| Gosner stage | 2 | 0.6553 | 1.2183 | 12 | 30 | 0.3162 |

**Table S4** Overall output for one-way multivariate analysis of variance (MANOVA) for Wright’s-stained blood smears sampled at Gosner stage 40. Proportions of each leukocyte were calculated per 3000 RBCs and numbers were log-transformed (log_10_[count+1]) to approximate normal distributions. The log-transformed proportion of each leukocyte type were the (six) response variables, and exposure group (control vs exposed) was the predictor variable.

| Variable | Df | Pillai’s Trace | Approx. F-statistic | Num Df | Den Df | P-value |
| --- | --- | --- | --- | --- | --- | --- |
| Exposure | 1 | 0.57546 | 0.67774 | 6 | 3 | 0.6871 |

**Table S5** Overall output for one-way multivariate analysis of variance (MANOVA) for Wright’s-stained blood smears sampled at Gosner stage 42. Proportions of each leukocyte were calculated per 3000 RBCs and numbers were log-transformed (log_10_[count+1]) to approximate normal distributions. The log-transformed proportion of each leukocyte type were the (six) response variables, and exposure group (control vs exposed) was the predictor variable.

| Variable | Df | Pillai’s Trace | Approx. F-statistic | Num Df | Den Df | P-value |
| --- | --- | --- | --- | --- | --- | --- |
| Exposure | 1 | 0.58535 | 0.47056 | 6 | 2 | 0.7994 |

**Table S6** Overall output for one-way multivariate analysis of variance (MANOVA) for Wright’s-stained blood smears sampled at Gosner stage 45. Proportions of each leukocyte were calculated per 3000 RBCs and numbers were log-transformed (log_10_[count+1]) to approximate normal distributions. The log-transformed proportion of each leukocyte type were the (six) response variables, and infection outcome (control, exposed-cleared and exposed-moribund) was the predictor variable. Bold values denote statistical significance (p<0.05).

| Variable | Df | Pillai’s Trace | Approx. F-statistic | Num Df | Den Df | P-value |
| --- | --- | --- | --- | --- | --- | --- |
| Outcome | 2 | 0.90152 | 2.5989 | 12 | 38 | **0.01248** |

**Table S7** Univariate statistics output for the one-way multivariate analysis of variance (MANOVA) for Wright’s-stained blood smears at Gosner stage 45. Proportions of each leukocyte were calculated per 3000 RBCs and numbers were log-transformed (log_10_[count+1]) to approximate normal distributions. The log-transformed proportion of each leukocyte type were the (six) response variables, and infection outcome (control, exposed-cleared and exposed-moribund) was the predictor variable. Bold values denote statistical significance (p<0.05).

| Monocyte | Df | Sum Sq | Mean Sq | F-value | P-value |
| --- | --- | --- | --- | --- | --- |
| Outcome | 2 | 0.4833 | 0.241649 | 3.85E+00 | **0.03624** |
| Residuals | 23 | 1.4453 | 0.062839 |  |  |

| Lymphocyte | Df | Sum Sq | Mean Sq | F-value | P-value |
| --- | --- | --- | --- | --- | --- |
| Outcome | 2 | 0.072281 | 0.036141 | 3.0173 | 0.0686 |
| Residuals | 23 | 0.275492 | 0.011978 |  |  |

| Neutrophil | Df | Sum Sq | Mean Sq | F-value | P-value |
| --- | --- | --- | --- | --- | --- |
| Outcome | 2 | 1.43045 | 0.71522 | 1.80E+01 | **1.96E-05** |
| Residuals | 23 | 0.91334 | 0.03971 |  |  |

| Basophil | Df | Sum Sq | Mean Sq | F-value | P-value |
| --- | --- | --- | --- | --- | --- |
| Outcome | 2 | 0.00987 | 0.004933 | 0.1474 | 0.8637 |
| Residuals | 23 | 0.76956 | 0.033459 |  |  |

| Eosinophil | Df | Sum Sq | Mean Sq | F-value | P-value |
| --- | --- | --- | --- | --- | --- |
| Outcome | 2 | 0.48788 | 0.24394 | 3.06E+00 | 0.06627 |
| Residuals | 23 | 1.8329 | 0.079691 |  |  |

| Thrombocyte | Df | Sum Sq | Mean Sq | F-value | P-value |
| --- | --- | --- | --- | --- | --- |
| Outcome | 2 | 0.1157 | 0.057872 | 2.80E-01 | 0.7581 |
| Residuals | 23 | 4.7485 | 0.206456 |  |  |

**Table S8** Linear regression models to assess the relationship between Bd load and individual leukocyte cell counts. Proportions of each leukocyte were calculated per 3000 RBCs and numbers were log-transformed (log_10_[count+1]) to approximate normal distributions. Bold values denote statistical significance (p<0.05).

| Monocyte | Sum Sq | Mean Sq | F-value | P-value |
| --- | --- | --- | --- | --- |
| (Intercept) | -3.591 | 1.836 | -1.956 | 0.06217 |
| Value | 5.516 | 1.806 | 3.054 | **0.00545** |

| Lymphocyte | Sum Sq | Mean Sq | F-value | P-value |
| --- | --- | --- | --- | --- |
| (Intercept) | 12.532 | 7.755 | 1.616 | 0.119 |
| Value | -6.685 | 4.823 | -1.386 | 0.178 |

| Neutrophil | Sum Sq | Mean Sq | F-value | P-value |
| --- | --- | --- | --- | --- |
| (Intercept) | -6.86 | 1.484 | -4.621 | 0.000109 |
| Value | 7.339 | 1.218 | 6.027 | **3.19E-06** |

| Basophil | Sum Sq | Mean Sq | F-value | P-value |
| --- | --- | --- | --- | --- |
| (Intercept) | -0.07147 | 3.71911 | -0.019 | 0.985 |
| Value | 1.70566 | 3.32978 | 0.512 | 0.613 |

| Eosinophil | Sum Sq | Mean Sq | F-value | P-value |
| --- | --- | --- | --- | --- |
| (Intercept) | 3.13 | 1.224 | 2.558 | 0.0173 |
| Value | -2.287 | 1.883 | -1.214 | 0.2365 |

| Thrombocyte | Sum Sq | Mean Sq | F-value | P-value |
| --- | --- | --- | --- | --- |
| (Intercept) | 3.2343 | 2.0317 | 1.592 | 0.124 |
| Value | -0.9682 | 1.3255 | -0.73 | 0.472 |

- 1. Toluidine Blue stained liver histology samples

**Table S9** Output of a one-way analysis of variance (ANOVA) for white blood cell (WBC) counts of Toluidine Blue stained liver histology samples from control animals. Log-transformed (log_10_[count+1]) white blood cell (WBC) abundance was the response variable, and Gosner stage (40, 42 and 45) was the predictor variable.

| WBC | Df | Sum Sq | Mean Sq | F-value | P-value |
| --- | --- | --- | --- | --- | --- |
| Stage | 2 | 8.004 | 4.002 | 3.51E+00 | 0.053 |
| Residuals | 17 | 19.394 | 1.141 |  |  |

**Table S10** Output of a one-way analysis of variance (ANOVA) for red blood cell (RBC) counts of Toluidine Blue stained liver histology samples from control animals. Red blood cell (RBC) abundance was the response variable, and Gosner stage (40, 42 and 45) was the predictor variable.

| RBC | Df | Sum Sq | Mean Sq | F-value | P-value |
| --- | --- | --- | --- | --- | --- |
| Stage | 2 | 541061 | 270531 | 9.69E-01 | 0.4 |
| Residuals | 17 | 4747048 | 279238 |  |  |

**Table S11** Output of a one-way analysis of variance (ANOVA) for macrophage counts of Toluidine Blue stained liver histology samples from control animals, with Tukey HSD post-hoc analyses. Macrophage abundance was the response variable, and Gosner stage (40, 42 and 45) was the predictor variable. Bold values denote statistical significance (p<0.05).

| Macrophage | Df | Sum Sq | Mean Sq | F-value | P-value |
| --- | --- | --- | --- | --- | --- |
| Stage | 2 | 18535610 | 9267805 | 15.28 | **0.000159** |
| Residuals | 17 | 10308203 | 606365 |  |  |

| Stage | Diff | Lower | Upper | P-value |
| --- | --- | --- | --- | --- |
| 42-40 | 126.8 | -1136.6115 | 1390.212 | 0.9641935 |
| 45-40 | 1986.7 | 892.5535 | 3080.846 | **0.0006267** |
| 45-42 | 1859.9 | 765.7535 | 2954.046 | **0.0011744** |

**Table S12** T-test comparing the white blood cell (WBC), red blood cell (RBC), and melanomacrophage counts of control and exposed animals at Gosner stage 40.

| t-test | Df | t value | P-value |
| --- | --- | --- | --- |
| WBC | 5.4183 | -2.0235 | 0.09455 |
| RBC | 7.0212 | 1.5348 | 0.1686 |
| Macrophage | 7.9913 | 0.82389 | 0.4339 |

**Table S13** T-test comparing the white blood cell (WBC), red blood cell (RBC), and melanomacrophage counts of control and exposed animals at Gosner stage 40.

| t-test | Df | t value | P-value |
| --- | --- | --- | --- |
| WBC | 4.9308 | -1.1447 | 0.3048 |
| RBC | 6.0083 | -0.80902 | 0.4493 |
| Macrophage | 3.7944 | -2.0707 | 0.1109 |

**Table S14** Output of a one-way analysis of variance (ANOVA) for log-transformed (log_10_[count+1]) white blood cell (WBC) counts of Toluidine Blue stained liver histology samples at Gosner stage 45, with Tukey HSD post-hoc analyses. Cell counts was the response variable, and infection outcome (control, exposed-cleared and exposed-moribund) was the predictor variable. Bold values denote statistical significance (p<0.05).

| WBC | Df | Sum Sq | Mean Sq | F-value | P-value |
| --- | --- | --- | --- | --- | --- |
| Outcome | 2 | 38.19 | 19.095 | 1.74E+01 | **1.81E-05** |
| Residuals | 25 | 27.38 | 1.095 |  |  |

| Mean comparison | Diff | Lower | Upper | P-value |
| --- | --- | --- | --- | --- |
| Exposed-Control | -1.623916 | -3.0515493 | -0.1962831 | **0.023557** |
| Moribund-Control | 1.513319 | 0.4169719 | 2.6096669 | **0.0056426** |
| Moribund-Exposed | 3.137236 | 1.7656101 | 4.5088611 | **0.0000181** |

**Table S15** Output of a one-way analysis of variance (ANOVA) for log-transformed (log_10_[count+1]) red blood cell (RBC) counts of Toluidine Blue stained liver histology samples at Gosner stage 45. Cell counts was the response variable, and infection outcome (control, exposed-cleared and exposed-moribund) was the predictor variable.

| RBC | Df | Sum Sq | Mean Sq | F-value | P-value |
| --- | --- | --- | --- | --- | --- |
| Outcome | 2 | 0.753 | 0.3763 | 2.48E+00 | 0.104 |
| Residuals | 25 | 3.801 | 0.152 |  |  |

**Table S16** Output of a one-way analysis of variance (ANOVA) for macrophage counts of Toluidine Blue stained liver histology samples at Gosner stage 45. Cell counts was the response variable, and infection outcome (control, exposed-cleared and exposed-moribund) was the predictor variable.

| Macrophage | Df | Sum Sq | Mean Sq | F-value | P-value |
| --- | --- | --- | --- | --- | --- |
| Outcome | 2 | 390949 | 195474 | 3.11E-01 | 0.735 |
| Residuals | 25 | 15699483 | 627979 |  |  |

**Table S17** Output of a one-way analysis of variance (ANOVA) for Toluidine Blue stained liver histology samples of control animals, with Tukey HSD post-hoc analyses. Macrophage density (square root) was the response variable, and Gosner stage (40, 42 and 45) was the predictor variable. Bold values denote statistical significance (p<0.05).

| Macrophage | Df | Sum Sq | Mean Sq | F-value | P-value |
| --- | --- | --- | --- | --- | --- |
| Stage | 2 | 375667 | 187833 | 28.44 | **3.77E-06** |
| Residuals | 17 | 112283 | 6605 |  |  |

| Mean comparison | Diff | Lower | Upper | P-value |
| --- | --- | --- | --- | --- |
| 42-40 | 52.77984 | -79.07943 | 184.6391 | 0.5706029 |
| 45-40 | 297.94188 | 183.74841 | 412.1354 | **0.0000108** |
| 45-42 | 245.16205 | 130.96857 | 359.3555 | **0.0001085** |

**Table S18** Output of a one-way analysis of variance (ANOVA) for Toluidine Blue stained liver histology samples at Gosner stage 45. Macrophage density (sum of cell areas) was the response variable, and infection outcome (control, exposed-cleared and exposed-moribund) was the predictor variable.

| Macrophage | Df | Sum Sq | Mean Sq | F-value | P-value |
| --- | --- | --- | --- | --- | --- |
| Outcome | 2 | 9.66E+09 | 4.83E+09 | 0.917 | 0.413 |
| Residuals | 24 | 1.27E+11 | 5.27E+09 |  |  |
